# Dysregulation, functional implications, and prognostic ability of the circadian clock across cancers

**DOI:** 10.1002/cam4.2035

**Published:** 2019-02-21

**Authors:** Zekun Liu, Kai Yu, Jian Zheng, Huan Lin, Qi Zhao, Xiaolong Zhang, Weiyi Feng, Liyu Wang, Jianjun Xu, Dawei Xie, Zhi‐Xiang Zuo, Ze‐Xian Liu, Qichang Zheng

**Affiliations:** ^1^ Department of Hepatobiliary Surgery Union Hospital, Tongji Medical College, Huazhong University of Science and Technology Wuhan China; ^2^ Sun Yat‐sen University Cancer Center State Key Laboratory of Oncology in South China, Collaborative Innovation Center for Cancer Medicine Guangzhou China; ^3^ The Second Affiliated Hospital of Guangzhou University of Chinese Medicine Guangzhou China; ^4^ Big Data Research Center, School of Computer Science and Engineering University of Electronic Science and Technology of China Chengdu China

**Keywords:** cancer biology, genomics, immunology, survival

## Abstract

It has been proposed that the circadian rhythm generally plays important roles in tumor suppression, but there is also evidence that disruption of the canonical circadian pathway has anticancer effects. In this study, we systematically analyzed the aberrances of circadian clock genes across cancers based on data from The Cancer Genome Atlas (TCGA). These data showed that the frequencies of mutations and copy number alterations in core clock genes (*PER1/2/3*, *CLOCK*, *CRY1/2,* and *ARNTL*) were low, but that the expression levels of core clock genes were downregulated by the higher levels of DNA methylation in most tumors. The circadian clock index (CCI) was established through a principal component analysis, and this measure well represents the overall expression of the core clock genes. In fact, the CCI was significantly lower in hepatocellular carcinoma with HBV infection than in other cancers. Furthermore, pathways such as the MAPK, JAK‐STAT, and immune‐related signaling pathways were enriched in tumors with high CCI values. Interestingly, the CCI was generally positively related to the immunophenoscores and immunophenotypes of tumors. Additionally, the expression levels of core clock genes and the CCI were also generally positively related to survival across cancers. Taken together, the results of this study provide a comprehensive analysis of circadian clock aberrances in cancer, and the results should aid further investigations of the molecular mechanisms of cancer and the development of therapeutic strategies.

## 1 BACKGROUND

The 2017 Nobel Prize in Physiology or Medicine was awarded to Jeffrey C. Hall, Michael Rosbash and Michael W. Young for their leading discoveries of the molecular mechanisms controlling the circadian rhythm.^1^, ^2^ The circadian clock is critical for the normal physiological functions of cells, and disruption of the circadian system has been proposed to pose an important cancer risk. Several recent studies have demonstrated that aberrances in the circadian rhythm are involved in various cancers, such as prostate cancer,^3^, ^4^ breast cancer,^5^ endometrial cancer,^6^ colorectal cancer,^7^, ^8^ liver cancer,^9^ lung cancer,^10^ and leukemia.^11^ For example, disruption of the circadian system could promote hepatocarcinogenesis through chronic jet lag‐driven gene dysregulation and liver metabolic dysfunction,^9^ decrease lung cancer survival, and promote lung tumor growth and progression.^10^ Sulli *et al* have recently shown that REV‐ERB agonists could serve as anticancer agents.^12^ Alternatively, Puram *et al* have found that disruption of the circadian pathway could exert antileukemic effects.^11^ Thus, an investigation of the circadian system could provide clues for improving the understanding of the molecular mechanisms underlying tumourigenesis and might provide helpful information for the development of cancer therapies.^13^, ^14^, ^15^, ^16^, ^17^, ^18^, ^19^

The circadian rhythm in humans is orchestrated by the autoregulatory transcription and translation feedback loops of core clock genes, which comprise the activator genes, including *CLOCK* and *ARNTL* (also known as *BMAL1*), and the repressor genes, including *PER1*, *PER2*, *CRY1,* and *CRY2*.^20^, ^21^ Basic helix‐loop‐helix (*bHLH*)‐*PER*‐*ARNT*‐*SIM* (PAS) transcription factors, *CLOCK* and its heterodimeric partner *ARNTL* form a complex that binds to the regulatory elements of core clock genes, including repressors (*PER1*, *PER2,* and *PER3*) and the cryptochrome (*CRY1* and *CRY2*),^20^, ^21^ which are also regulated by the E3 ligase complex of SKP1‐cullin‐F‐box protein‐β‐TrCP (SCF‐β‐TrCP)‐based ubiquitination.^20^, ^21^ In addition, the nuclear receptors *NR1D1* (*REV‐ERBα*) and *NR1D2* (*REV‐ERBβ*), which are regulated by *CLOCK* and *ARNTL*, could be driven by *RORα* and *RORβ* to repress the transcription of *ARNTL* and *NFIL3*, whereas *NFIL3* could, in turn, repress D‐box‐binding protein (DBP) to regulate ROR nuclear receptors.^20^, ^21^ Through comprehensive bioinformatics analyses, Lehmann *et al* recently constructed a regulatory network for the mammalian circadian clock.^22^ Thus, the orchestration of the circadian clock is complicated and involves many genes. In general, various genes, including *CLOCK*, *ARNTL*, *PER1*/2/3, and *CRY1*/*2*, form the core components of the mammalian circadian clock (CCMCCs), and these establish complicated molecular circuits that orchestrate the different phases of the circadian rhythm.^20^, ^21^ Furthermore, the expression of a substantial fraction (~5%‐20%) of genes is under the control of the circadian rhythm.^20^, ^21^ According to the Circadian Gene Database (CGDB),^23^ nearly 2000 genes show rhythmic expression, and many of these have been implicated to play roles in cancer.

Krugluger *et al* have found that the downregulation of *PER1* is correlated with high‐grade colon tumors,^24^ and Huisman *et al* have revealed that the circadian rhythm is disrupted in colorectal liver metastases.^7^ Through a population‐based case‐control study, Zhu *et al* have demonstrated that SNPs in core clock genes are significantly associated with susceptibility to prostate cancer.^25^ In addition, melatonin can resynchronize the dysregulated circadian rhythm in prostate cancer cells and should thus be investigated as an agent for the treatment of prostate cancer.^26^ Altered expression of core clock genes was also observed in chronic myeloid leukemia,^27^ and both malignant and normal hematopoietic cells harbor an intact clock and undergo robust circadian oscillations.^11^ Methylome profiling in human hepatocellular carcinomas (HCCs) has revealed that *PER3* is hypermethylated,^28^ and the overexpression of *PER1* due to inhibition of miR‐34a decreases the growth of cholangiocarcinomas.^29^ Hypermethylation‐triggered epigenetic inactivation of *ARNTL* has been detected in hematologic malignancies,^30^ and the overexpression of *ARNTL* could increase oxaliplatin sensitivity in colorectal cancer.^31^ Relogio *et al* have found that *RAS* can deregulate the mammalian circadian clock^32^ and revealed a remarkable interplay between the circadian clock and pre‐mRNA splicing in cancer.^33^ Recently, Shilts *et al* have performed comprehensive analyses that indicated widespread dysregulation of the circadian clock in cancer,^34^ and Ye *et al* have observed that most circadian clock genes are related to survival, oncogenic pathways, and anticancer drug sensitivity.^35^ Based on these findings, El‐Athman and Relogio have proposed that escaping circadian regulation might be an emerging hallmark of cancer.^36^ Taken together, the results strongly indicate that the rhythm orchestrated by the circadian clock is involved in tumourigenesis, cancer progression, and metastases, and detailed systematic analyses of circadian clock genes and rhythmic genes in cancer should be performed at different molecular levels.

In this study, we systematically analyzed the molecular dysregulation, functional implications, and clinical relevance of the circadian clock across 20 cancers based on data from The Cancer Genome Atlas (TCGA). The results revealed that the circadian clock genes were most often dysregulated through DNA hypermethylation‐based disruption of expression rather than mutations or copy number alterations. Based on the principal component analysis, we established the circadian clock index to represent the overall expression of core circadian clock genes and discovered that it is closely related to viral infections, various signaling pathways, such as the MAPK, JAK‐STAT, immune‐related, protein export, nucleotide excision repair, mismatch repair and cell cycle pathways, immunophenotypes, and survival. Our study provided a comprehensive analysis of the role of the circadian clock in cancer, and the results should be helpful for further investigations of circadian clock‐related molecular mechanisms and the development of therapies for cancer.

## 2 METHODS

### 2.1 Gene set curation and data obtainment

The circadian rhythm‐related genes were curated from a previous systematic review.^21^ The core clock genes include *CLOCK*, *ARNTL*, *PER1*, *PER2*, *PER3*, *CRY1,* and *CRY2,* 20, 21 whereas the CCMCCs are more extensive and contain 22 genes, including the core clock genes plus *BTRC*, *CSNK1D*, *CSNK1E*, *CUL1*, *DBP*, *FBXL21*, *FBXL3*, *NFIL3*, *NR1D1*, *NR1D2*, *PRKAA1*, *PRKAA2*, *RORA*, *RORB*, and *SKP1.* 20, 21 A gene list of 1350 rhythmic genes was downloaded from the CGDB, which claims that the transcript‐level oscillations of these genes have been validated in previous publications through various methods, such as RT‐PCR, northern blot, and in situ hybridization.^23^ In general, core clock genes and CCMCCs are the key orchestrators and important regulators of the circadian rhythm, respectively,^21^ and rhythmic genes are regulated by the circadian rhythm.^23^ All the data on mutations, copy number variations, DNA methylation, expression, and clinical outcomes were obtained from a level 3 dataset from TCGA at FireBrowse (http://gdac.broadinstitute.org, 2016 January). The analyses were performed using genomic and transcriptome data from both tumor and normal tissues for the following 20 types of cancer: bladder urothelial carcinoma (BLCA), breast invasive carcinoma (BRCA), cholangiocarcinoma (CHOL), colon adenocarcinoma (COAD), cervical and endocervical cancers (CESCs), esophageal carcinoma (ESCA), glioblastoma multiforme (GBM), head and neck squamous cell carcinoma (HNSC), kidney chromophobe (KICH), kidney renal clear cell carcinoma (KIRC), kidney renal papillary cell carcinoma (KIRP), liver hepatocellular carcinoma (LIHC), lung adenocarcinoma (LUAD), lung squamous cell carcinoma (LUSC), pancreatic adenocarcinoma (PAAD), prostate adenocarcinoma (PRAD), rectum adenocarcinoma (READ), stomach adenocarcinoma (STAD), thyroid carcinoma (THCA), and uterine corpus endometrial carcinoma (UCEC). The somatic copy‐number alterations (SCNAs) of the core clock genes were obtained from cBioPortal,^37^, ^38^ and immunophenotype data were obtained from The Cancer Immunome Atlas (TCIA, https://tcia.at/) with substantial assistance from the authors.^39^

### 2.2 Analysis of somatic mutations, driver genes, copy numbers, and DNA methylation

Mutation data were obtained from the downloaded MAF files, and only nonsilent mutations were reserved for the analysis. The frequency ratio was calculated as the mutation frequency divided by the mutation burden to eliminate the diversity of mutation burden among cancers. A frequently mutated gene (FMG) in one type of cancer was identified based on a mutation frequency greater than 5% in that type of cancer, whereas the enrichment of FMGs in the set of rhythmic genes was performed based on hypergeometric statistics. Driver gene analysis for each cancer type was conducted using MutSigCV v1.2 with the GenePattern online server (https://genepattern.broadinstitute.org)^40^ with a cutoff of *P* < 0.05. The correlation between mutations of 375 driver genes^40^ and the CCI was analyzed by regression method, while cancer types and tumor mutation burden were adjusted. The *P* values across 375 genes were corrected for multiple hypothesis testing using method of Benjamini & Hochberg, and only genes under 10% false discovery rate (FDR) were considered as significantly mutated. Genes and promoter regions were annotated with the Bioconductor (Release 3.7) R package IlluminaHumanMethylation450kanno.ilmn12.hg19 (version 0.6.0) for DNA methylation 450 K data. Only the methylation sites within the promotor regions of the core clock genes were considered in the differential methylation analysis, which was performed with Wilcoxon rank sum tests.

### 2.3 Analysis of gene expression

Normalized RNA‐Seq v2 data were downloaded for the gene expression analysis and are summarized in Table 1. The R package DESeq2 was employed to perform differential expression analysis between tumors and normal tissues,^41^ and the genes with adjusted *P* < 0.05 were defined as differentially expressed. The principal component analysis (PCA) was conducted with R packages.^42^ The principal component 1 (PC1) was employed as the circadian clock index (CCI) to represent the overall expression of the core clock genes, and the CCI values were performed with Wilcoxon rank sum tests. To test whether the mutational status of the driver genes^40^, ^43^ was significantly associated with the CCI, rank‐transformed CCI was modeled via linear regression as a function of the gene's mutational status, and the rank‐transformed mutation burden was used to diminish confounding effects. The gene set enrichment analysis (GSEA) was carried out using GSEA software^44^ with default parameters to study differences in expression of the circadian clock genes between tumor and normal tissues and between high‐ and low‐grade tumors and to investigate the pathway differences between tumors with high (top 25%) and low (bottom 25%) CCI scores. The GSEA results were visualized with gseapy package (https://pypi.python.org/pypi/gseapy), and the other visualizations were generated with ggplot2^45^ and other R packages such as pheatmap and ComplexHeatmap.^42^

**Table 1:** The number of samples and abbreviations for the 20 types of cancers investigated in this study

Cancer type	Abbreviation	Number of samples
Bladder urothelial carcinoma	BLCA	412
Breast invasive carcinoma	BRCA	1098
Cervical and endocervical cancers	CESC	307
Cholangiocarcinoma	CHOL	51
Colon adenocarcinoma	COAD	460
Esophageal carcinoma	ESCA	185
Glioblastoma multiforme	GBM	613
Head and Neck squamous cell carcinoma	HNSC	528
Kidney Chromophobe	KICH	113
Kidney renal clear cell carcinoma	KIRC	537
Kidney renal papillary cell carcinoma	KIRP	323
Liver hepatocellular carcinoma	LIHC	377
Lung adenocarcinoma	LUAD	585
Lung squamous cell carcinoma	LUSC	504
Pancreatic adenocarcinoma	PAAD	185
Prostate adenocarcinoma	PRAD	499
Rectum adenocarcinoma	READ	171
Stomach adenocarcinoma	STAD	443
Thyroid carcinoma	THCA	503
Uterine Corpus Endometrial Carcinoma	UCEC	560

## 3 RESULTS

### 3.1 Molecular alterations of the circadian rhythm across cancers

**Figure 1:**
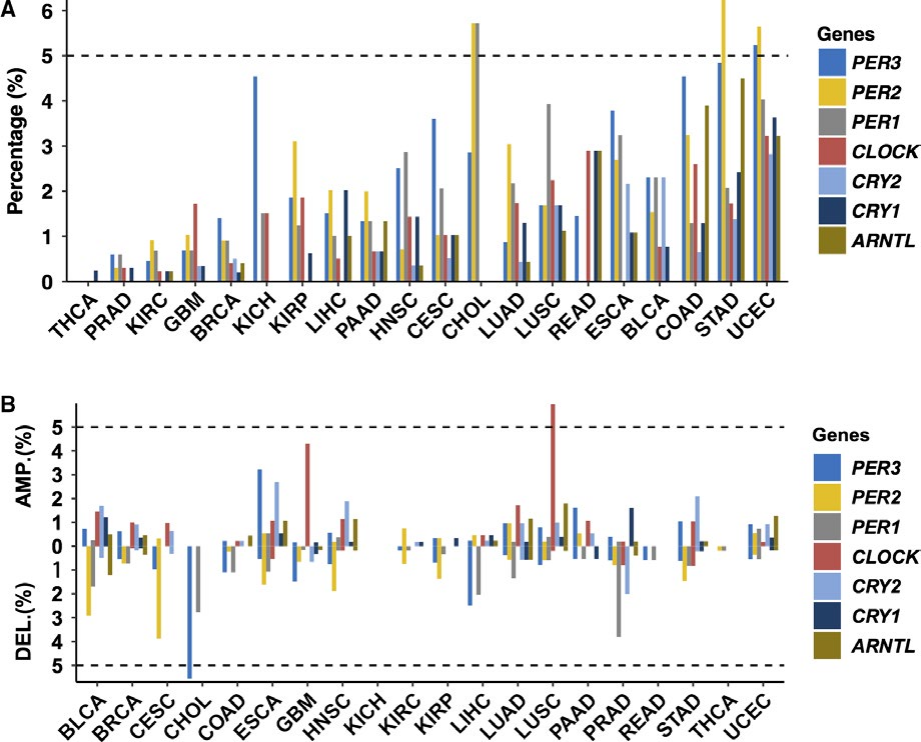
The genomic alterations of CCMCCs across cancers. (A) Mutation proportions of the core clock genes in tumor samples. (B) Proportions of somatic copy number alterations of the core clock genes, including amplification (AMP.) and deletion (DEL.) in tumor samples

Since the circadian rhythm is critical for the physiological functioning of organs, we analyzed the molecular alterations of circadian clock genes. As shown in Figure 1A, the mutation frequencies of core clock genes varied notably among cancers. However, the mutation frequencies of almost all core clock genes were lower than 5%, with the following exceptions: *PER2* in CHOL, STAD and UCEC, *PER1* in CHOL, and *PER3* in UCEC. Thus, since the mutation rates of core clock genes were found to be low, the circadian rhythm system might not be disrupted by mutation in cancers. In general, *PER1/2/3* exhibited the highest mutation frequencies among the core clock genes. In fact, the mutation‐based driver gene analysis showed that only *PER3* might be a potential driver gene in CESC and ESCA. Furthermore, the mutation rates of other CCMCCs in cancer were also low (Figure S1 and S8). To further dissect the genomic alterations of circadian rhythm, the copy number variations of core clock genes were also analyzed (Figure 1B), and the results showed that the copy number alterations of core clock genes were limited, although both amplifications and deletions of core clock genes were observed. The alteration frequencies of core clock genes were lower than 5% in most cancers, with the exception of *PER3* in CHOL and *CLOCK* in LUSC. Taken together, the core clock genes appear to be relatively stable at the genomic level.

To investigate aberrances in rhythmic genes across cancers, the mutations in the 1350 experimentally identified rhythmic genes curated from the CGDB were systematically analyzed in various cancers. The genes with mutation frequencies greater than 5% were defined as FMGs, and enrichment analyses were then performed across cancers based on hypergeometric test. The results revealed that FMGs were enriched in rhythmic genes in cancers, including COAD (*P* = 0.0343), ESCA (*P* = 0.0339), KIRC (*P* = 0.0060), and UCEC (*P* = 0.0074), which indicated that rhythmic genes have relatively high mutation rates in these cancers. Furthermore, the results obtained using MutSigCV software showed that a number of rhythmic genes were driver genes in these cancers, although rhythmic genes were not significantly enriched with driver genes. For example, more than 10% of driver genes were also circadian genes in KIRP and THCA. Thus, rhythmic genes might be increasingly involved in rhythmic dynamics and could be preferentially mutated in several cancers.

### 3.2 Differential expression of circadian clock genes across cancers

**Figure 2:**
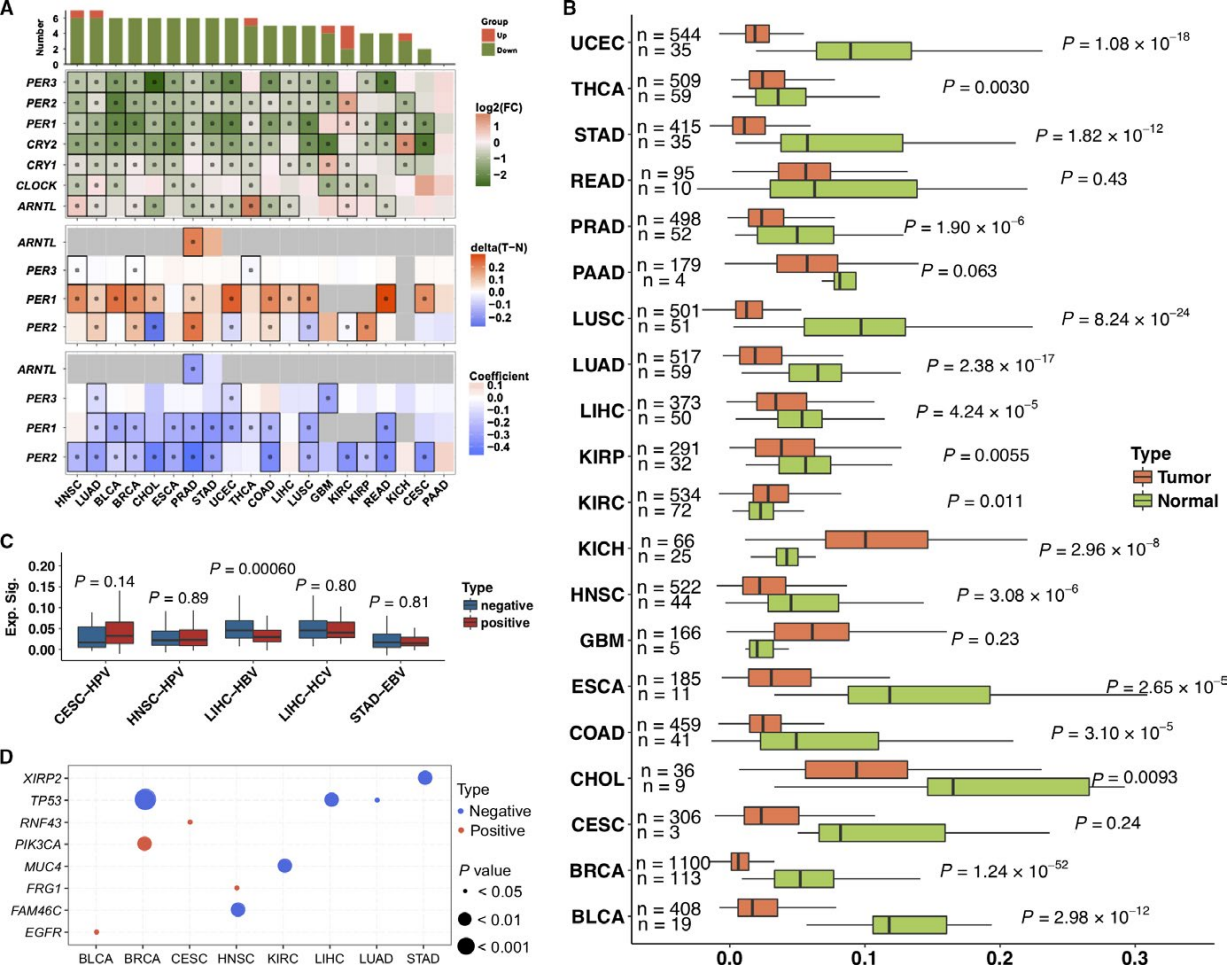
Differential expression and methylation of the core clock genes. (A) Top panel: the log2‐transformed fold changes (tumor vs. normal) are shown to indicate the differential expression of the core clock genes between tumor and normal tissues. Middle panel: beta value differences (tumor minus normal) are presented to show the differential methylation of core clock genes between tumor and normal tissues. Bottom panel: the coefficients for correlations between the expression and methylation of the core clock genes are shown. The dots represent statistically significant results (*P *< 0.05). (B) Differences in the CCI between tumor and normal tissues. (C) Differences in the CCI between virus‐infected tumor tissues (positive) and uninfected tumor tissues (negative). (D) The correlation between mutations of driver genes and the CCI

The circadian rhythm has been generally proposed as a tumor‐suppressive mechanism that is disrupted in cancers,^14^ the low frequencies of genomic alterations in core clock genes indicate that the circadian clock might be dysregulated at the gene expression level. The differential expression analysis of the core clock genes across cancers was performed with DESeq2 software, and the significant results (adjusted *P* < 0.05) are shown in Figure 2A. It was clear that the expression levels of core clock genes were significantly downregulated in almost all cancers. A limited number of exceptions were observed, and these included the overexpression of *PER1/2* in KIRC, *ARNTL* in KRIC and HNSC, THCA and CRY2 in KICH, and *CLOCK* in CESC and LUAD. Thus, the expression levels of core clock genes are generally downregulated in tumors. Since there is no timepoint information for the samples in TCGA, the observed differences in expression should be considered as the differences between tumor and normal tissues. Furthermore, we performed a GSEA with the core clock genes among cancers, and the results shown in Figure S2 reveal that the core clock genes were significantly downregulated in seven cancers, namely, UCEC, BRCA, HNSC, BLCA, CESC, STAD, and GBM.

To dissect the potential cause of the low expression of the core clock genes in cancers, we analyzed the DNA methylation status of the promoters of the core clock genes. DNA methylation data were available only for *PER1/2/3* and *ARNTL*, and the analysis showed that the promoter regions of *PER1/2/3* and *ARNTL* were generally highly methylated (Figure 2A) in most cancers and that the expression level and methylation status were highly correlated. Thus, this high degree of methylation might be the major cause of the low expression of *PER1/2/3* and *ARNTL* in tumors. In addition, the low expression of other CCMCCs was consistent with their hypermethylation in most cancers (Figure S3A). Furthermore, we established the circadian clock index (CCI) for core clock genes based on the PCA. According to the results shown in Figure S3B, all the proportions of PC1 in 27 cancers were greater than 66%, and the proportions in READ, GBM, COAD, UCEC, PRAD, BRCA, and CHOL were higher than 85%. Thus, the expression of core clock genes could be well represented by PC1, which indicated that PC1 could be employed as the CCI. The differences between tumor and normal tissues were then analyzed. As shown in Figure 2B, the CCI was generally significantly lower in almost all tumors, which was consistent with the differential expression analysis results. Because viral infection might contribute to aberrant gene expression, we analyzed the correlation between the CCI and viral infections. The results presented in Figure 2C show that the CCI was significantly lower in hepatitis B virus (HBV)‐infected liver tumors than in normal tissues, which indicate that HBV infection might contribute to the dysregulation of the circadian rhythm in liver cancer. Furthermore, we analyzed the correlation between the CCI and driver gene mutations through regression analysis. As the results shown in Figure 2D, the CCI was negatively correlated with mutations of driver genes including *TP53* in BRCA, LIHC, and LUAD, *XIRP2* in STAD, *MUC4* in KIRC and *FAM46C* in HNSC, while negatively correlations were observed for *RNF43* in CESC, *PIK3CA* in BRCA, *FRG1* in HNSC, and *EGFR* in BLCA.

Furthermore, the expression and methylation of CCMCCs were analyzed, and the results presented in Figure S8 show that the expression of CCMCCs was disrupted in tumors and that hypermethylation contributed substantially to this disruption. In tumor tissues, dysregulated circadian rhythm would disrupt the oscillatory expression of circadian genes; therefore, it was necessary to investigate the differential expression of rhythmic genes. DESeq2 was employed to identify the differentially expressed genes (DEGs) in 20 cancers using an adjusted *P* value less than 0.05 as the cutoff value. Enrichment analyses of DEGs in rhythmic genes across cancers were based on using a hypergeometric test. It was observed that DEGs were enriched in rhythmic genes in various cancers, including GBM (*P* = 7.87E‐07), CHOL (*P* = 0.0021), PAAD (*P* = 0.0024), and CESC (*P* = 0.0142), and thus indicated that disruption of the circadian rhythm might strongly influence gene expression in these cancers. Although the regulation of gene expression is complicated, the disruption of oscillatory expression in cancer is likely to be related to the dysregulation of gene expression in tumors.

### 3.3 Signaling pathways correlated with the disrupted circadian rhythm

**Figure 3:**
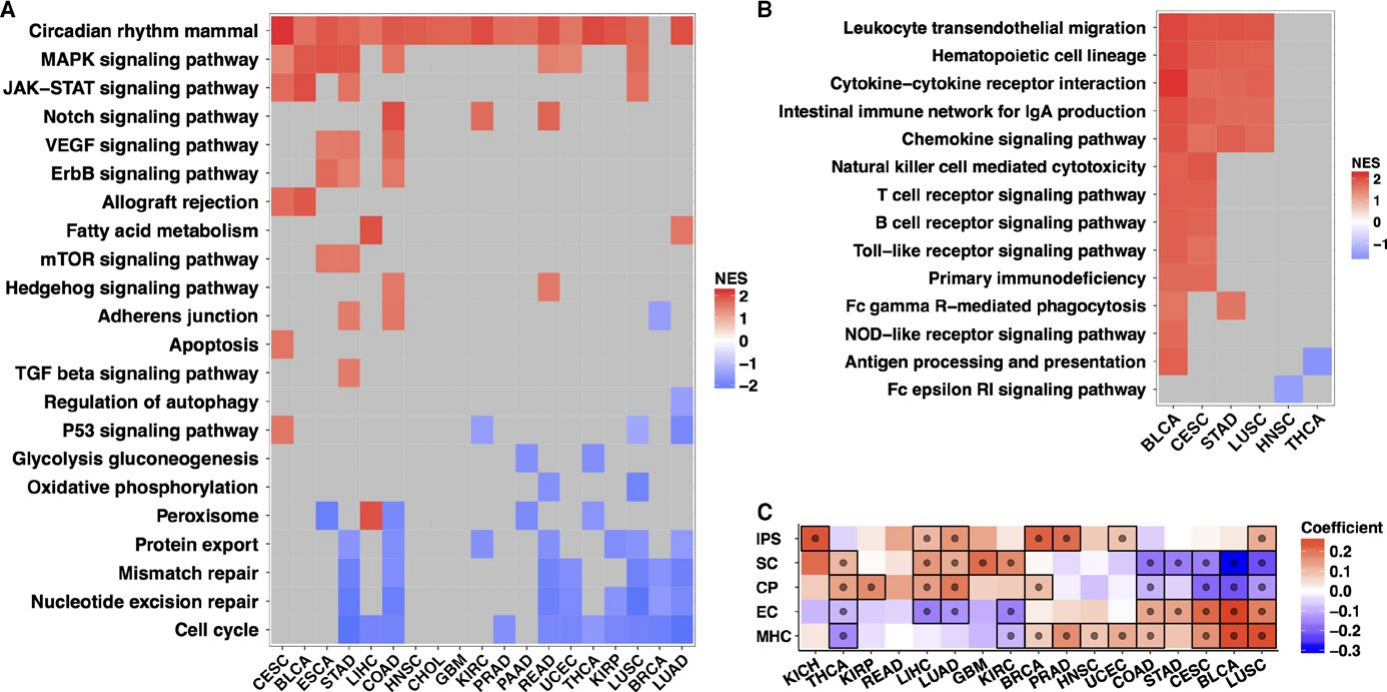
Relationships between the CCI, signaling pathways, and immunophenotypes. (A) Results of the GSEA of signaling pathways between high‐ and low‐CGI tumor tissues. NES is the normalized enrichment score in the GSEA. (B) Results of the GSEA of the immune‐related signaling pathways between high‐ and low‐CGI tumor tissues. (C) Correlations between immunophenotypes and the CCI; dots indicate statistically significant results (*P* < 0.05)

Since circadian rhythm is critical for normal physiological functioning and is disrupted in tumors, we analyzed the signaling pathways correlated with a disrupted circadian rhythm for the results might be helpful for future studies aiming to understand underlying molecular mechanisms and functions of the circadian rhythm in cancer. GSEA of signaling pathways was performed between high‐ and low‐CCI tumors (Figure 3A‐B, Figure S4). As shown in Figure 3A, various pathways were up‐ or downregulated in high‐CGI tumors compared with low‐CGI tumors. The mammalian circadian rhythm pathway was significantly upregulated in almost all high‐CGI tumors, with the exception of BRCA, and these findings provide further evidence showing that the CCI can accurately represent the circadian rhythm. Pathways such as the MAPK, JAK‐STAT, VEGF, ErbB, and Notch signaling pathways were positively correlated with circadian rhythm, whereas protein export, mismatch repair, nucleotide excision repair, and cell cycle were negatively related to circadian rhythm. Furthermore, CCI was positively correlated with a large number of metabolic pathways in LIHC (Figure S4A), which was consistent with the findings of previous studies.^46^, ^47^, ^48^, ^49^, ^50^

The circadian rhythm recently emerged as an important regulator of the immune system.^51^, ^52^ The GSEA results showed that a number of immune pathways were differentially activated in high‐ and low‐CCI tumor tissues (Figure 3B). For example, leukocyte transendothelial migration, hematopoietic cell lineage, cytokine‐cytokine receptor interaction, intestinal immune network for IgA production, and chemokine signaling pathway were upregulated in high‐CCI tumor tissues but not in low‐CCI tumor tissues in BLCA, CESC, STAD, and LUSC. Various pathways, including natural killer cell‐mediated cytotoxicity, T‐ and B‐cell receptor signaling pathways, Toll−like receptor signaling pathway, and primary immunodeficiency, were upregulated in high‐CCI tumor tissues compared with low‐CCI tumor tissues in BLCA and CESC. Fc gamma R‐mediated phagocytosis was upregulated in high‐CCI tumor tissues compared with low‐CCI tumor tissues in BLCA and STAD. The NOD‐like receptor signaling pathway was upregulated in high‐CCI tumor tissues compared with low‐CCI tumor tissues in BLCA. In BLCA and THCA, antigen processing and presentation were upregulated and downregulated in high‐CCI tumor tissues compared with low‐CCI tumor tissues, respectively. The Fc epsilon RI signaling pathway was downregulated in high‐CCI tumor tissues compared with low‐CCI tumor tissues in HNSC. Thus, the status of the circadian clock might be correlated with the activation of immune pathways in tumor tissues in a number of cancers.

Furthermore, we attempted to systematically investigate the relationship between the CCI and immunophenotypes based on previous immunogenomic analyses.^39^ As shown in Figure 3C, in various cancers, including KICH, LIHC, LUAD, BRCA, PRAD, UCEC, and LUSC, the CCI was significantly positively correlated with the immunophenoscore (IPS), which represents the immunogenicity of the tumor.^39^ The correlations between the CCI and four categories, namely, suppressor cells (including Tregs and MDSCs, SC), checkpoint (CP), effector cells (including activated CD8+ T cells, CD4+ T cells, Tem CD8+, and Tem CD4+ cells, EC) and HLAs (MHC) were analyzed (Figure 3C). We also observed that CCI was positively correlated with the repression of signals, including those to SC and CP in THCA, KIRP, LIHC, LUAD, GBM, and KIRC, but negatively correlated with the repression of signaling in COAD, STAD, CESC, BLCA, and LUSC. In contrast, the CCI was negatively correlated with the activation of signaling, including signals to EC and MHC, in THCA, LIHC, LUAD, and KIRC, but positively associated with the activation of signaling in PRAD, HNSC, UCEC, COAD, STAD, CESC, BLCA, and LUSC. Unexpectedly, the CCI was positively correlated with both CP and MHC in BRCA. Thus, the relationship between the circadian clock and immune signaling might be close but complicated, and more studies should be conducted to elucidate this network.

Furthermore, based on estimations of the abundances of immune cell types by CIBERSORT, the correlations between the CCI and immune cell types were analyzed, and the results are shown in Figure S5. The correlations were generally miscellaneous, while there were also special patterns that could be detected. For example, the CCI was positively correlated with resting mast cell, naïve B cell, resting memory CD4 T cell, and CD8 cell in most cancers and negatively correlated with macrophage M1, resting dendritic cell, follicular helper cell, and regulatory T cell in most cancers. The CCI was extremely positively correlated with activated dendritic cell in GBM and LIHC, activated mast cell in PRAD, plasma cell in GBM, and resting memory CD4 T cell in LIHC, and these correlations were either weaker or negative in other cancers. Strongly negative correlations were observed between the CCI and macrophage M0 in LIHC, macrophage M1 in KIRP, and neutrophil in KICH. Taken together, these lines of evidence show that the circadian rhythm system is strongly correlated with the immune status, but the relationship between circadian rhythm and immunophenotypes is complicated, and further investigations are needed in these cancers.

### 3.4 Clinical relevance of the circadian rhythm across cancers

**Figure 4:**
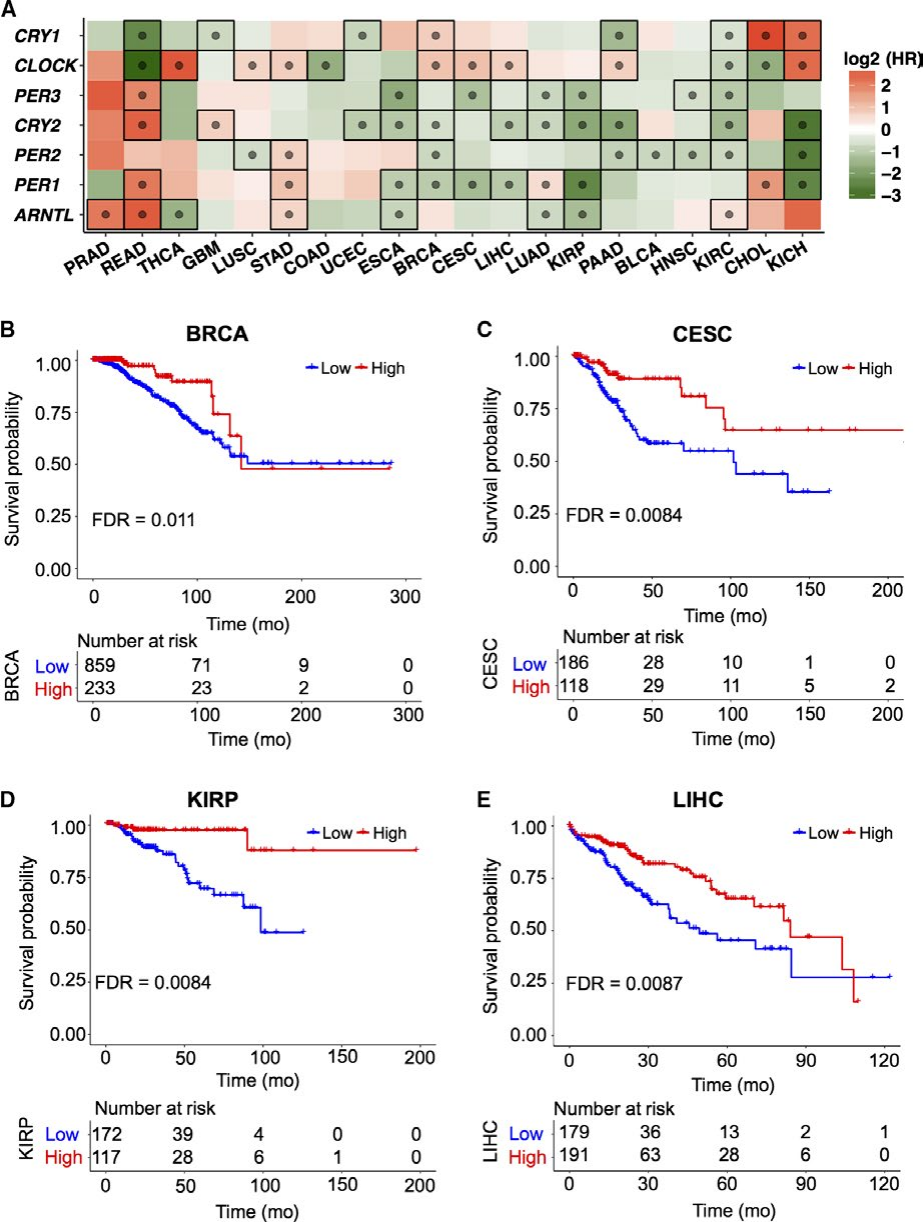
Prognostic ability of the core clock genes and the CCI across cancers. (A) Results of the Kaplan‐Meier analysis of overall survival according to expression of the core clock genes. The boxes containing dots indicate statistically significant results (*P* < 0.05). HR indicates the hazard ratio. (B‐E) Kaplan‐Meier analysis of overall survival according to the CCI in BRCA (B), CESC (C), KIRP (D), and LIHC (E)

A previous study found that the circadian rhythm is associated with survival in colorectal cancer patients.^8^ For circadian rhythm is crucial to cell physiology and the core clock genes were dysregulated at mutation, SCNA, DNA methylation and expression levels, the prognostic ability of the circadian system in cancers should be investigated. Survival analyses were performed based on the expression of the core clock genes with the best cutoff values across cancers. As summarized in Figure 4A, high expression levels of most core clock genes indicated low hazard ratio (HR) in most cancers except PRAD and STAD, whereas *CLOCK* was negatively correlated with survival in most cancers. Thus, it is obvious that high expression levels of the core clock genes generally predict better survival among cancers. The exceptions might be related to the multifunctional roles of core clock genes and need further investigation. Furthermore, the prognostic ability of the CCI was also studied. The survival analysis with the best cutoff value showed that a high CCI predicted better survival in BRCA, CESC, KIRP, and LIHC (Figure 4B‐E). Besides, we carefully analyzed relationships between circadian and clinical characteristics including smoking, alcohol, BMI, age, and tumor stages. It was observed that cigarette smoking was positively correlated with CCI in ESCA (Pearson r = 0.24, *P* = 0.02) and LUSC (*r* = 0.10, *P* = 0.04), while BMI was negatively correlated with CCI in READ (*r* = 0.25, *P* = 0.03). Furthermore, positive correlation was observed between age and THCA (*r* = −0.10, *P* = 0.03). However, no significant association between the CCI and alcohol was observed across cancers. Furthermore, we analyzed the correlation among tumor stages and circadian rhythm through GSEA of the core clock genes between high‐ and low‐stage tumors among cancers and found that the downregulated core clock genes were significantly enriched in high‐stage tumors in KICH and KIRC (Figure S6).

To further reveal the prognostic ability of the circadian clock genes, the survival analysis was performed among the CCMCCs. As shown in Figure S6, most CCMCCs were positively related with survival among cancers, although miscellaneous results were also observed. For example, in KIRC, high expression level of *FBXL3* predicted better survival, while *RORB*, *CSNK1D,* and *CSNK1E* significantly predicted poor survival (Figure S7). However, high expression of *FBXL3* was related to poor survival in STAD (Figure S7). Thus, the functional roles of CCMCCs in cancer survival should be further investigated.

## 4 DISCUSSION

Although it has long been known that the circadian rhythm plays crucial roles in physiology, this system had not attracted much attention until recently.^1^, ^2^ Common sense, epidemiological evidence, and biomedical studies all indicate that circadian rhythm and its orchestrating circadian clock genes are associated with cancer.^3^, ^4^, ^5^, ^6^, ^7^, ^8^, ^9^, ^10^, ^11^, ^12^, ^13^, ^14^, ^15^, ^16^, ^17^, ^18^, ^19^, ^24^, ^25^, ^26^, ^27^, ^28^, ^29^, ^30^, ^31^, ^34^ In this study, we systematically analyzed the dysregulation of circadian clock genes at different molecular levels across 20 types of cancer. Circadian clock genes were dysregulated at the expression level, which agreed with the findings of another recent comprehensive study.^34^ In general, the expression of circadian clock genes was downregulated in tumor tissues and correlated with their hypermethylation. Currently, the TCGA data have no timepoint information, which makes it unclear whether different timepoints could influence the expression of core clock genes in tumor tissues. The principal component analysis revealed that the CCI could well represent the expression of circadian clock genes, and the CCI was correlated with viral infection, various signaling pathways, immunophenotypes, and cancer patient survival.

Previously, Taniguchi *et. al.* reported that in hematologic malignancies *ARNTL* was transcriptionally silenced by the hypermethylation of its promoter CpG island.^30^ TCGA data showed that the expression of *ARNTL* was significantly lower in most solid tumors than in nontumor tissues; in addition, hypermethylation of *ARNTL* was also observed in PRAD (Figure 2A) and STAD, while the methylation status of *ARNTL* in other cancers was not available. Furthermore, hypermethylation of *PER1*/*PER2*/*PER3* was observed in most cancers, although the hypermethylation of *PER3* was only statistically significant in HNSC, BRCA, and THCA. Thus, the main cause of the lower expression levels of circadian clock genes might be hypermethylation of *ARNTL* and *PER1*/*PER2*/*PER3*, while the mechanisms for *CLOCK* and *CRY1/CRY2* require further investigation.

In general, the proportions of PC1 were sufficiently high to represent the expression of the core clock genes in PCA (Figure S3A). However, *CRY1* in GBM, *PER1*/*PER2* in KIRC, and *CRY2* in KICH had significantly higher expression levels in tumors than in nontumor tissue, and the CCI was also higher in tumors (Figure 2B). Thus, aberrances of the circadian clock in these three cancers should be more carefully studied. Interestingly, *PER1*/*PER2,* and *CRY2* are negative arms of the circadian feedback loop, while the role of *CRY1* is unclear.^20^, ^21^ The clock correlation distance model proposed by Shilts *et al* might be helpful for further analyses of aberrances of the circadian clock in these three cancers. Furthermore, this study clarified that the CCIs of the core clock genes were closely correlated with immunophenotypes and cancer patient survival. However, the relationships were not consistently positive or negative among cancer types. Thus, further detailed investigations should be performed to determine the mechanisms and functions of the dysregulation of the circadian clock in different cancer types.

## 5 CONCLUSIONS

The circadian rhythm and its regulatory circadian clock genes play crucial roles in cancer, and further investigation could advance the understanding of the functional roles of the circadian clock and the circadian rhythm in cancer and aid the development of potential therapies.

## CONFLICT OF INTEREST

The authors declare that they have no competing interests.
