# Supplementary figures and images for: Dysregulation, functional implications, and prognostic ability of the circadian clock across cancers
Source: Cancer Med. 2019 Feb 21;8(4):1710–20. doi: 10.1002/cam4.2035 (PMC6488113; doi:10.1002/cam4.2035)

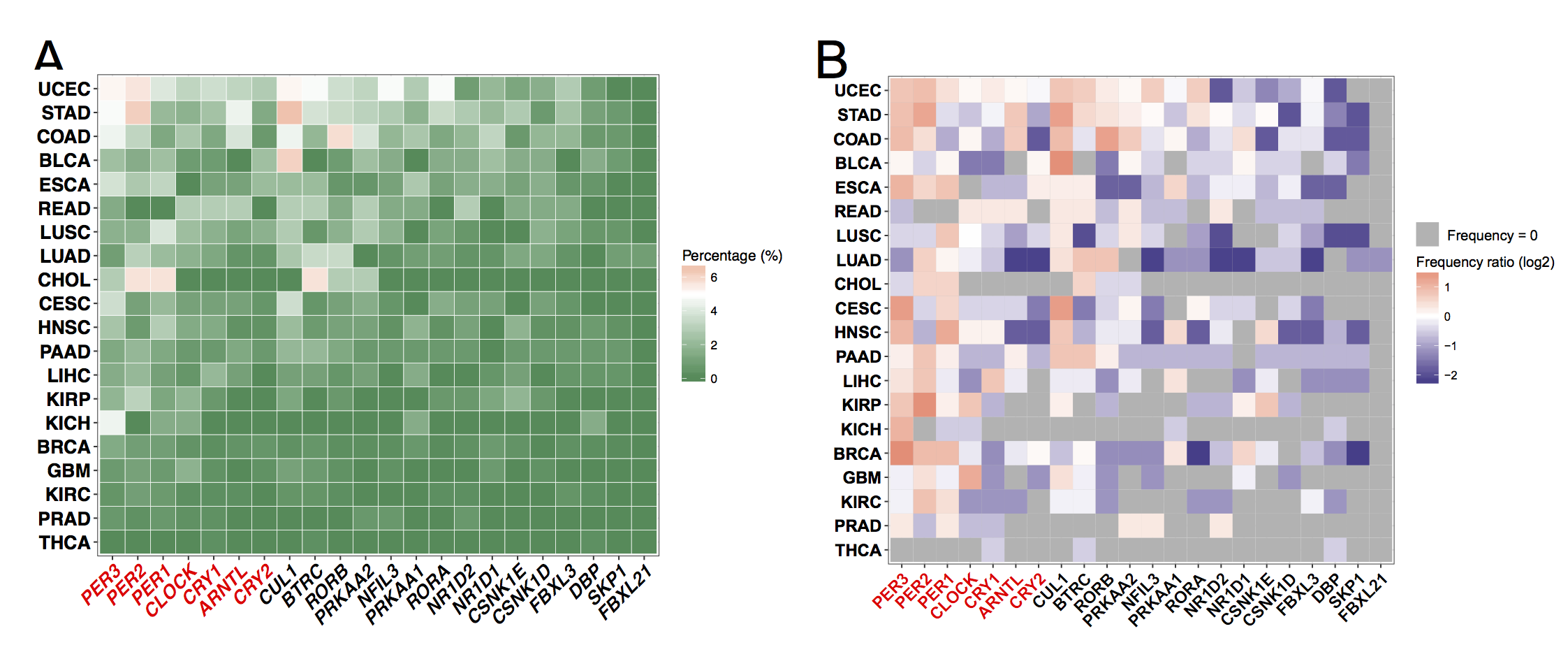

Supplement: Supplementary file 1 [file CAM4-8-1710-s001.tiff]

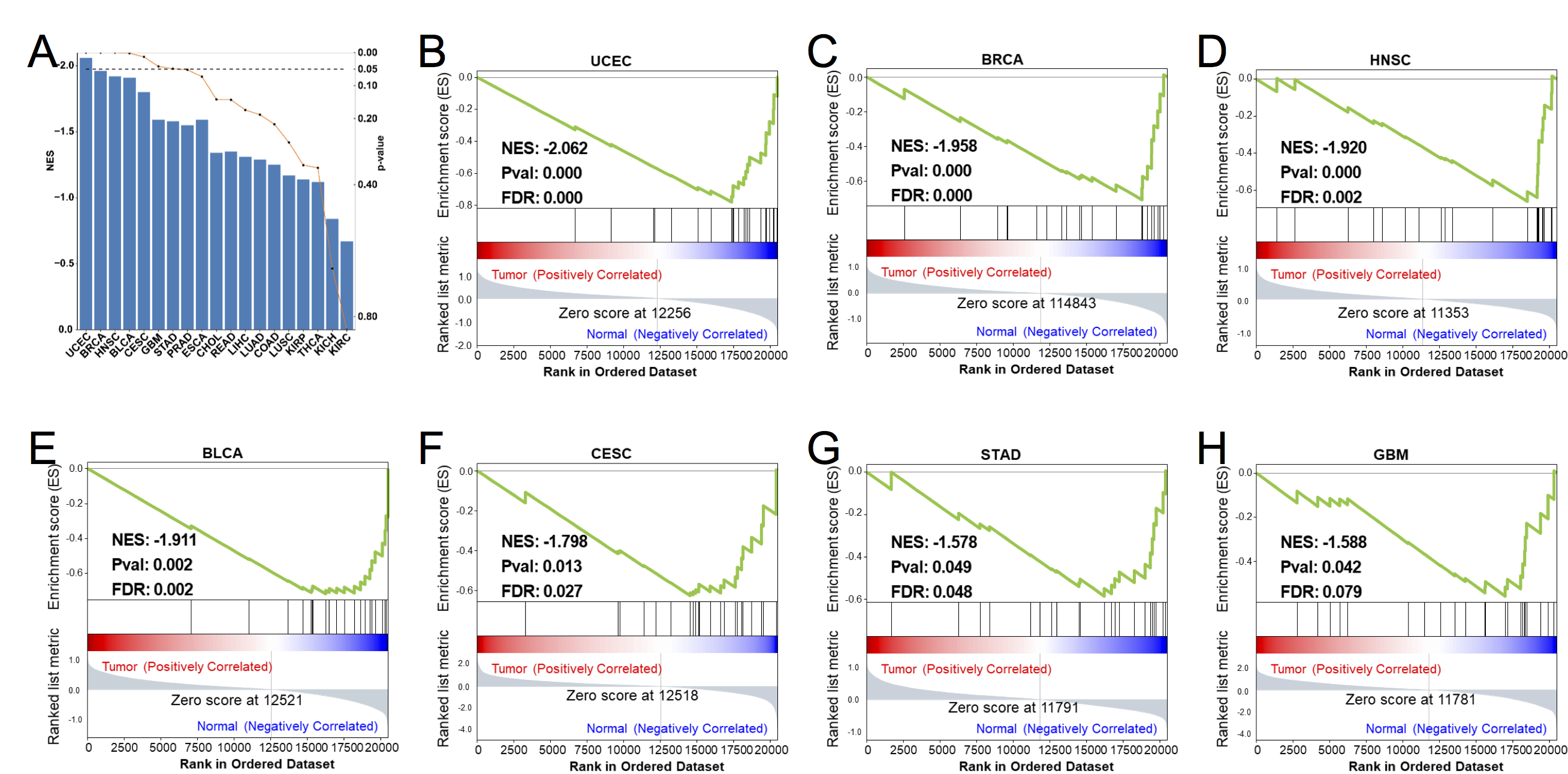

Supplement: Supplementary file 2 [file CAM4-8-1710-s002.tiff]

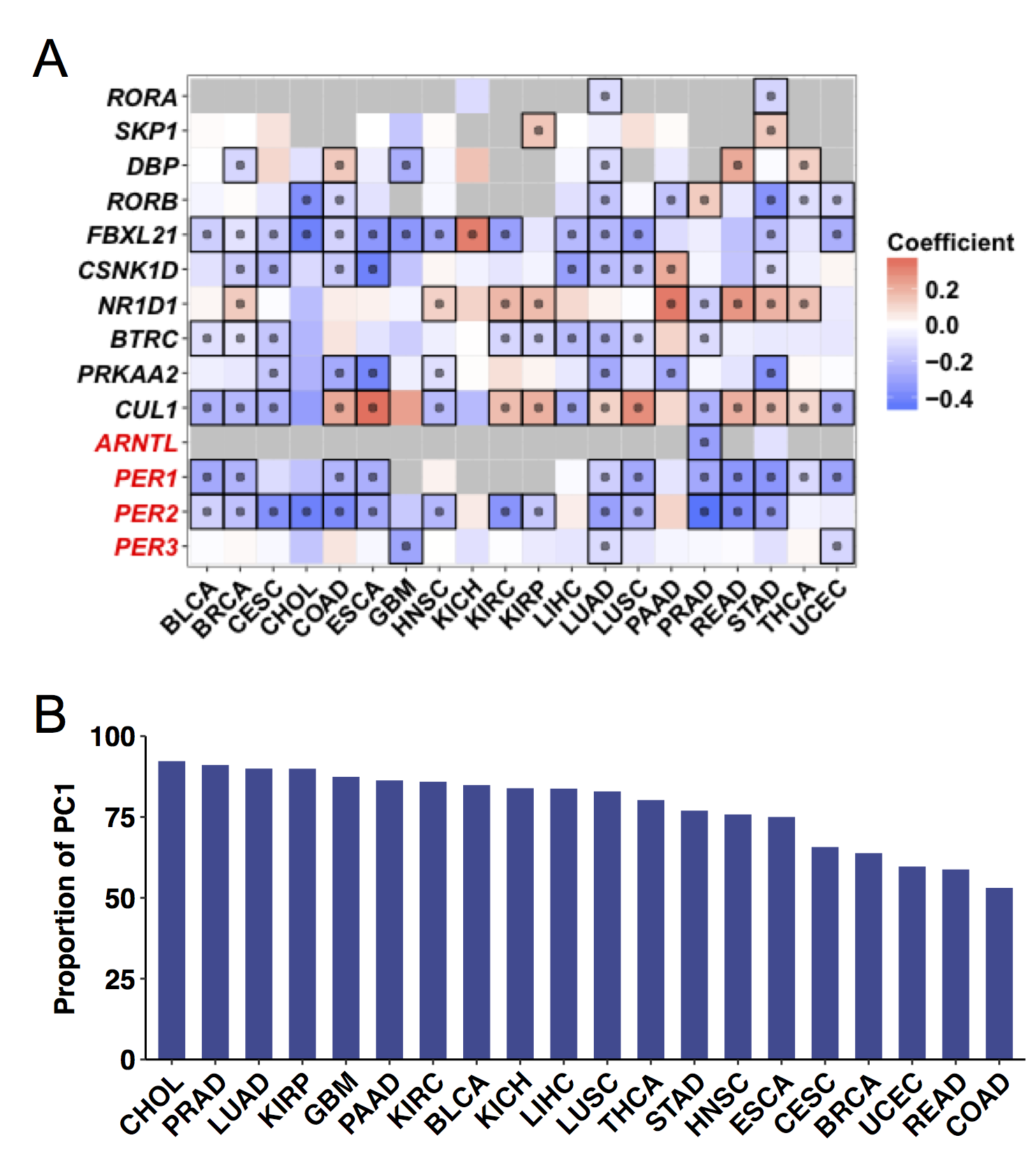

Supplement: Supplementary file 3 [file CAM4-8-1710-s003.tiff]

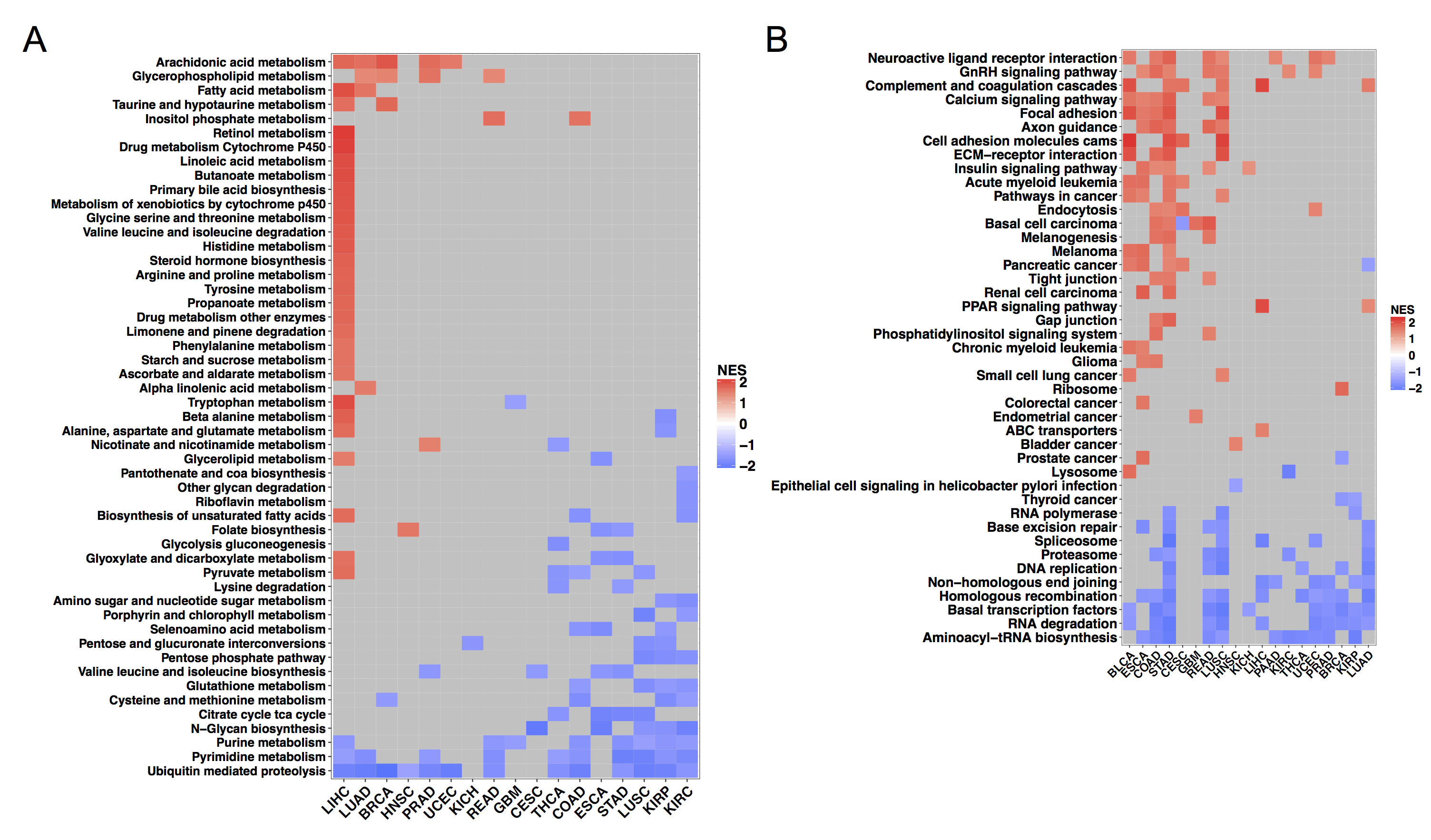

Supplement: Supplementary file 4 [file CAM4-8-1710-s004.tiff]

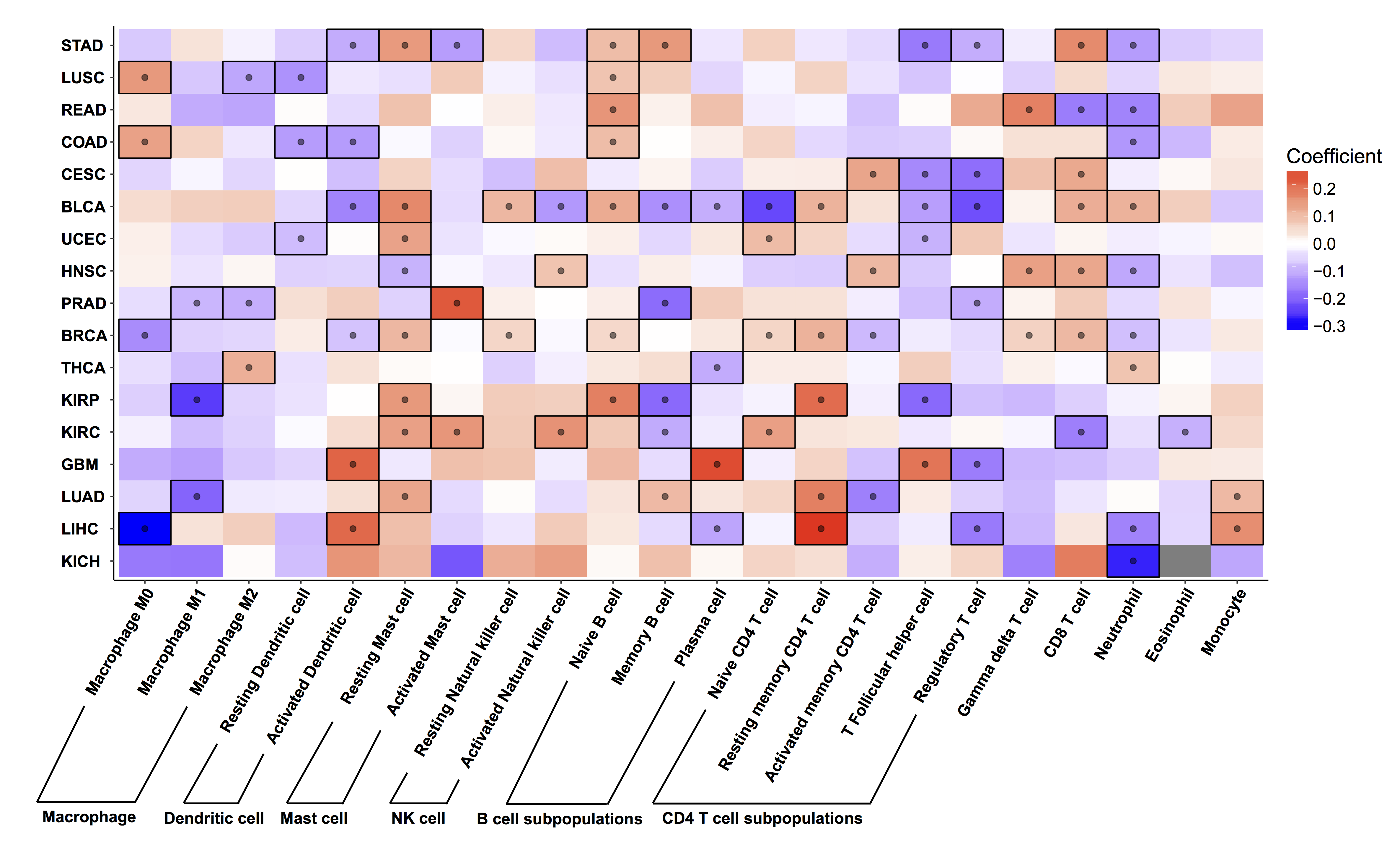

Supplement: Supplementary file 5 [file CAM4-8-1710-s005.tiff]

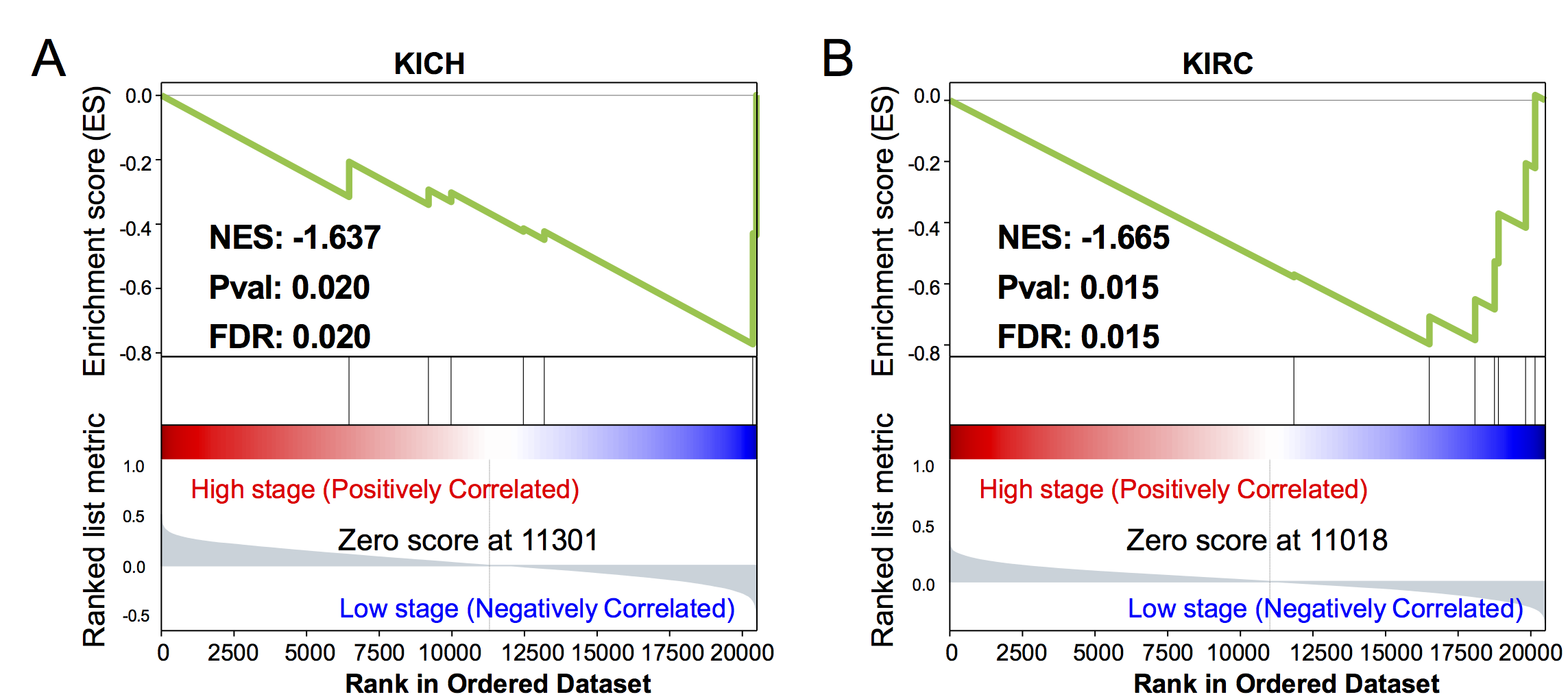

Supplement: Supplementary file 6 [file CAM4-8-1710-s006.tiff]

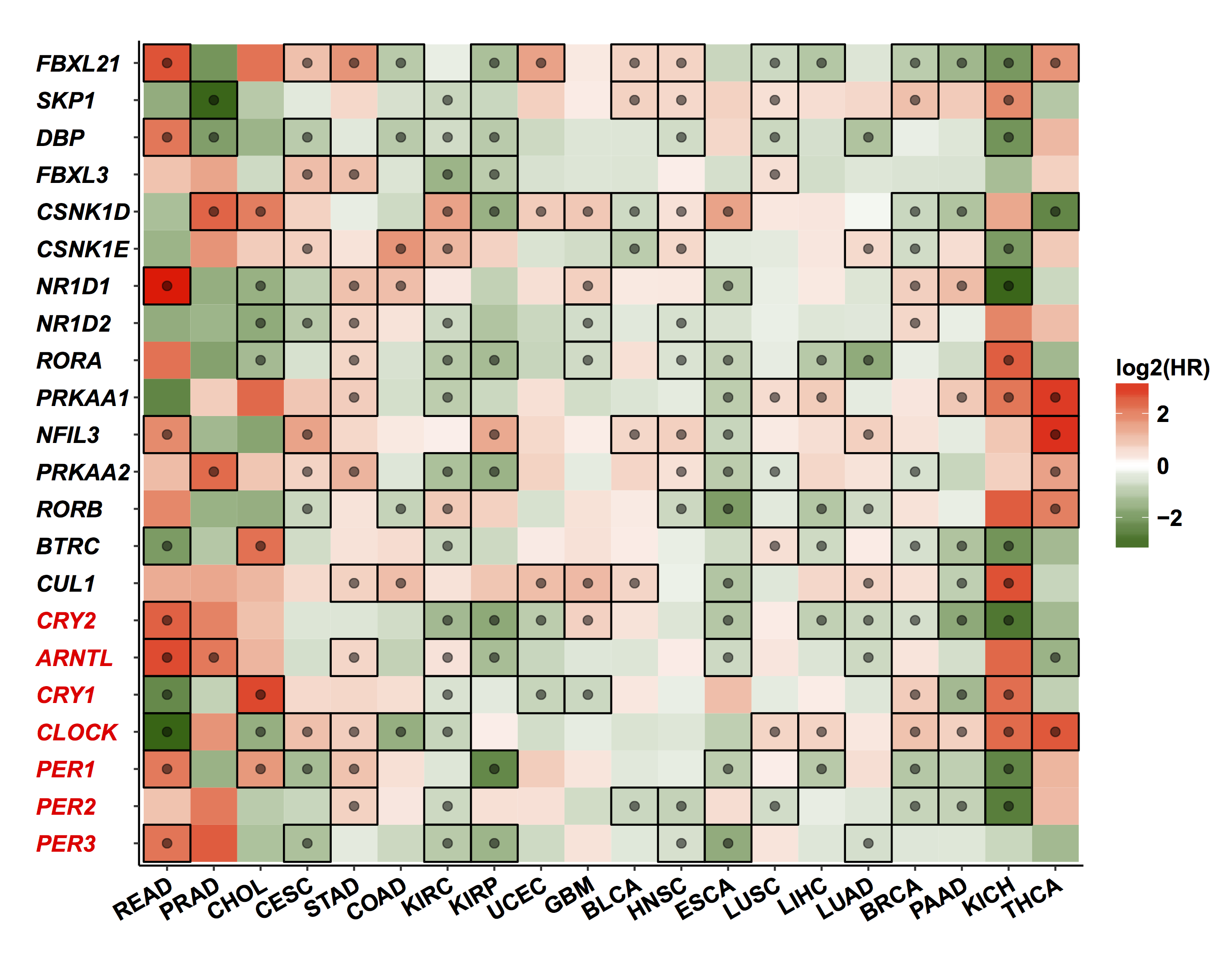

Supplement: Supplementary file 7 [file CAM4-8-1710-s007.tiff]
