# Supplementary material for: Dysregulation, functional implications, and prognostic ability of the circadian clock across cancers
Source: Cancer Med. 2019 Feb 21;8(4):1710–20. doi: 10.1002/cam4.2035 (PMC6488113; doi:10.1002/cam4.2035)

Figure S8A - Bladder urothelial carcinoma (BLCA)

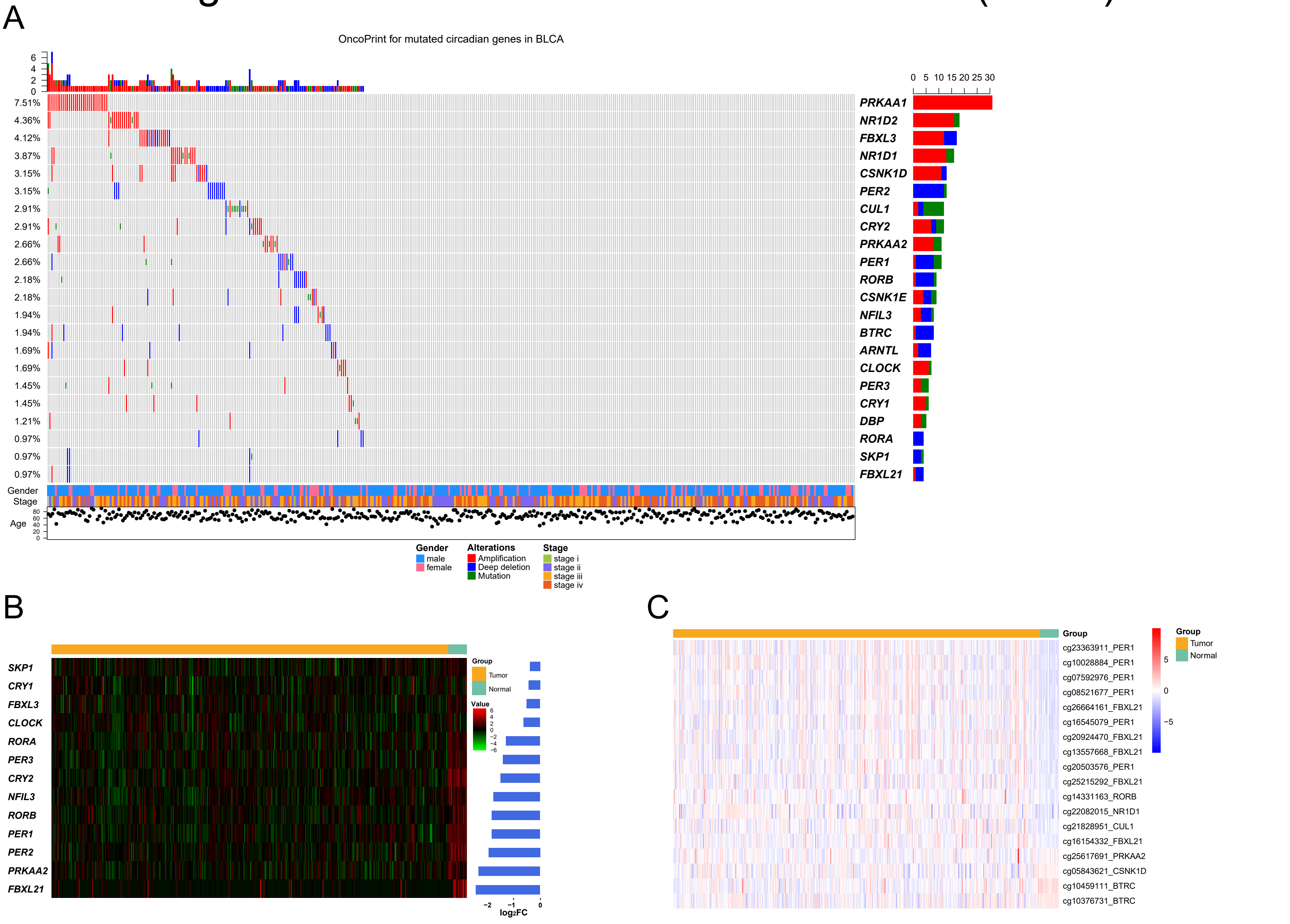

Figure S8B - Breast invasive carcinoma (BRCA)

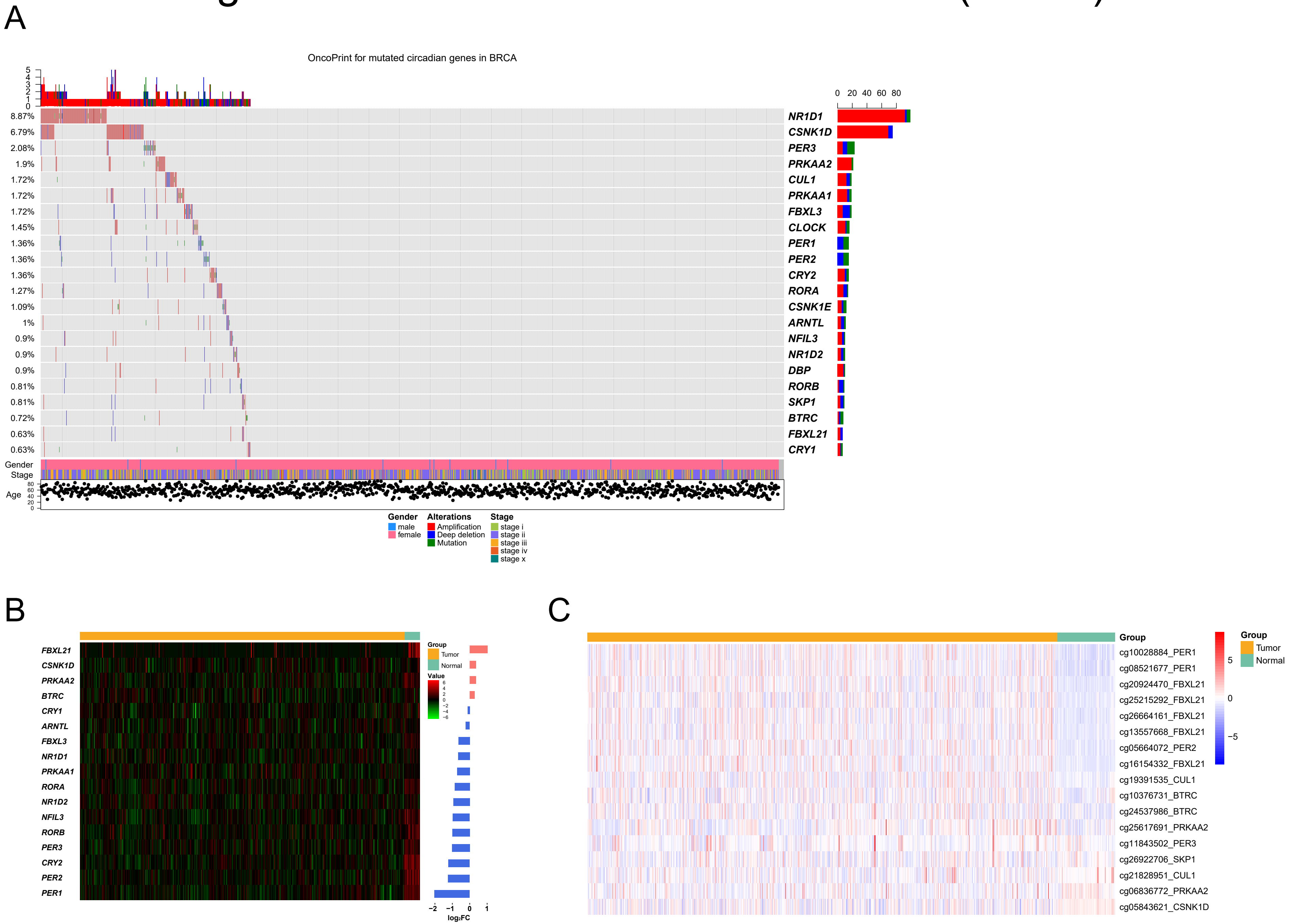

Figure S8C - Cervical and endocervical cancers (CESC)

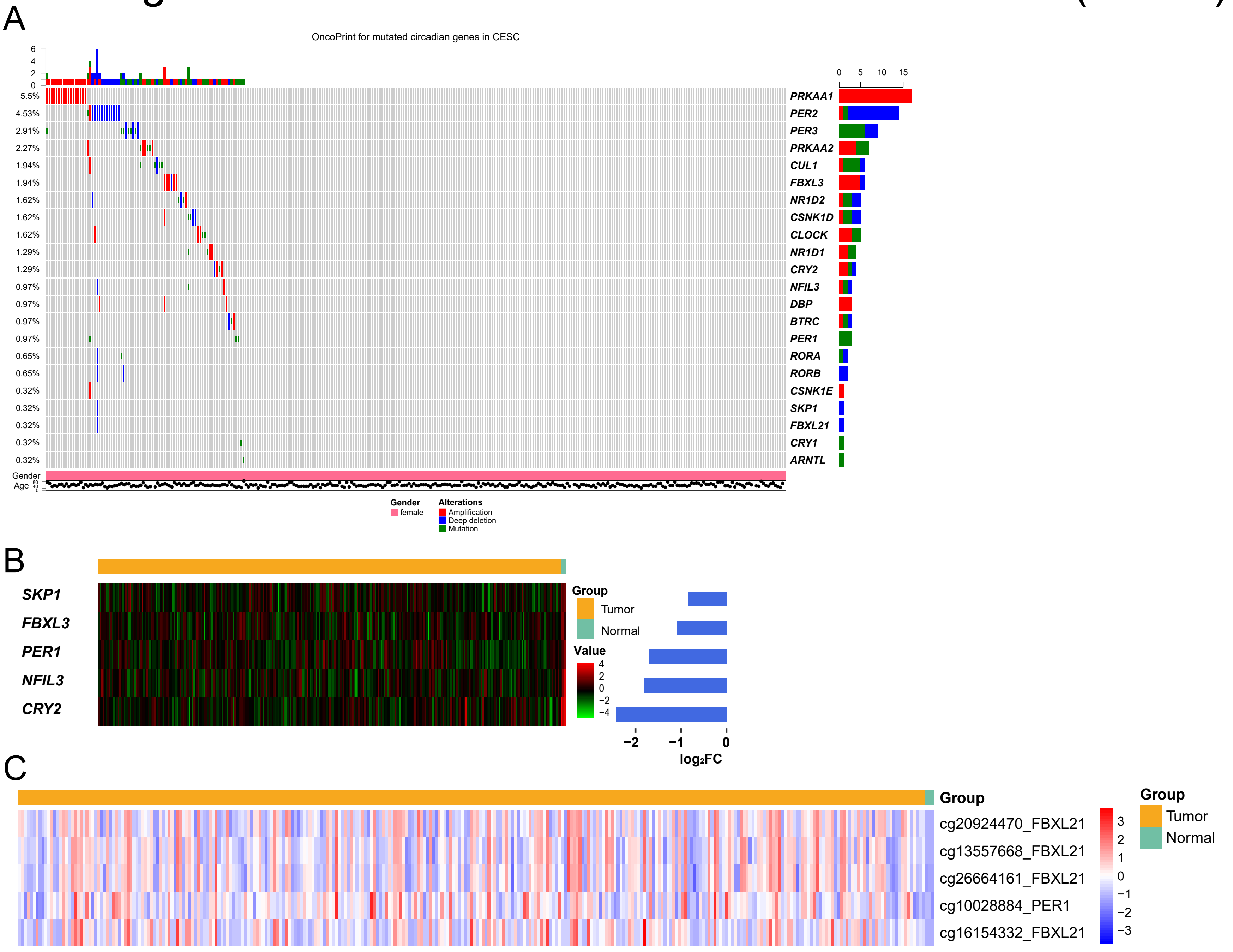

Figure S8D - Cholangiocarcinoma (CHOL)

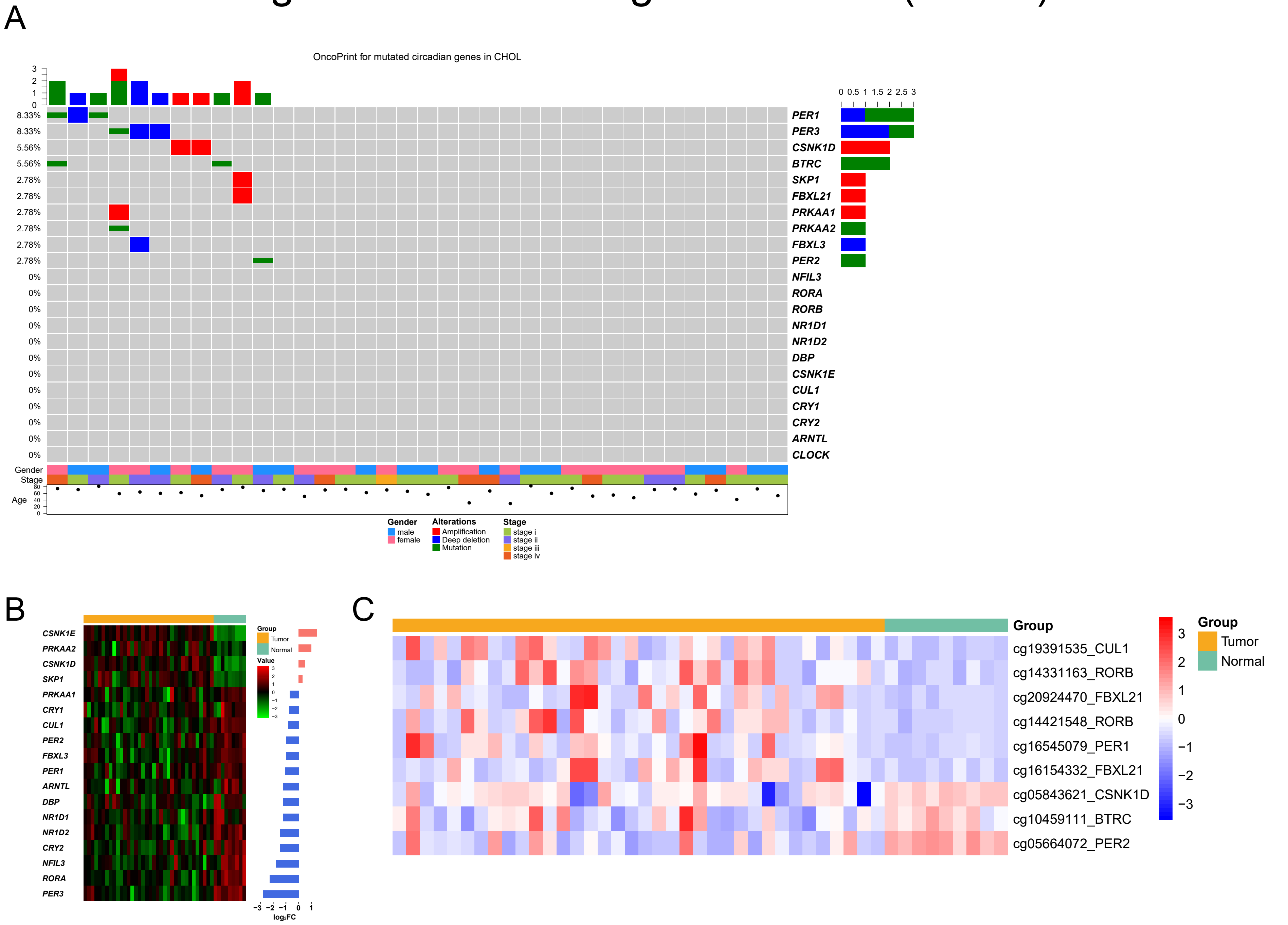

Figure S8E - Colon adenocarcinoma (COAD)

A

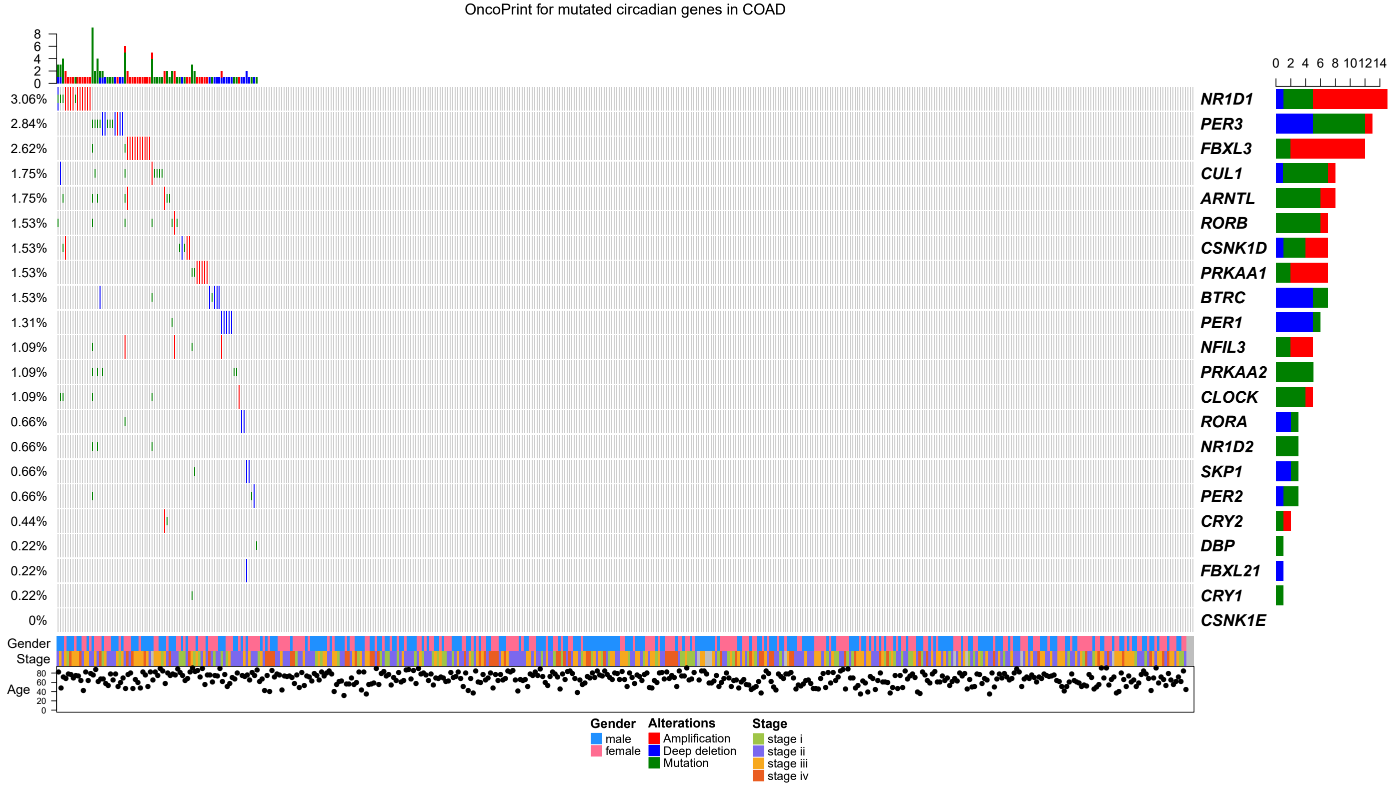

B

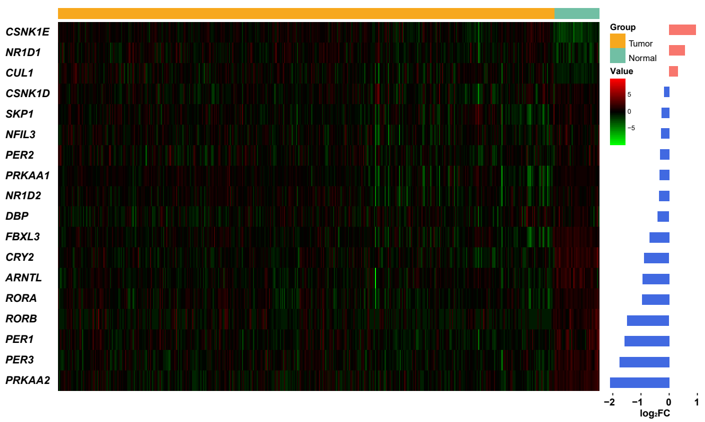

C

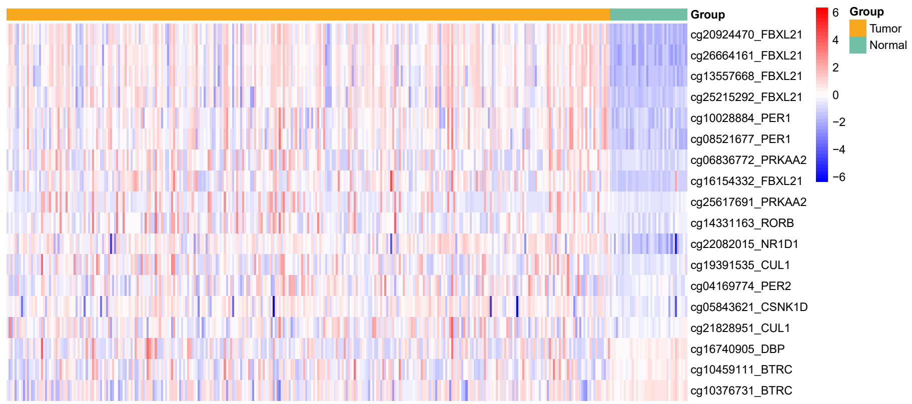

Figure S8F - Esophageal carcinoma (ESCA)

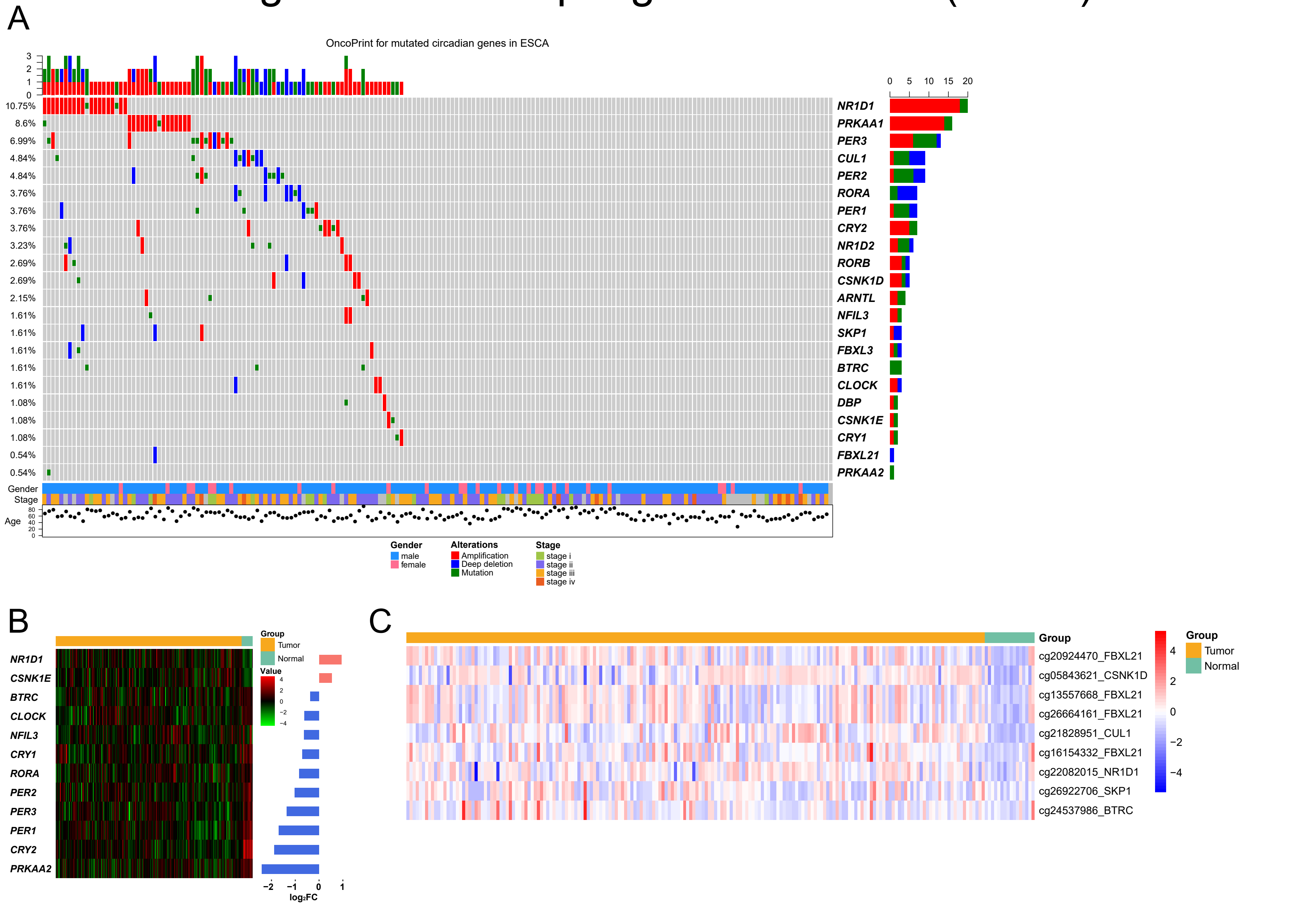

Figure S8G - Glioblastoma multiforme (GBM)

A

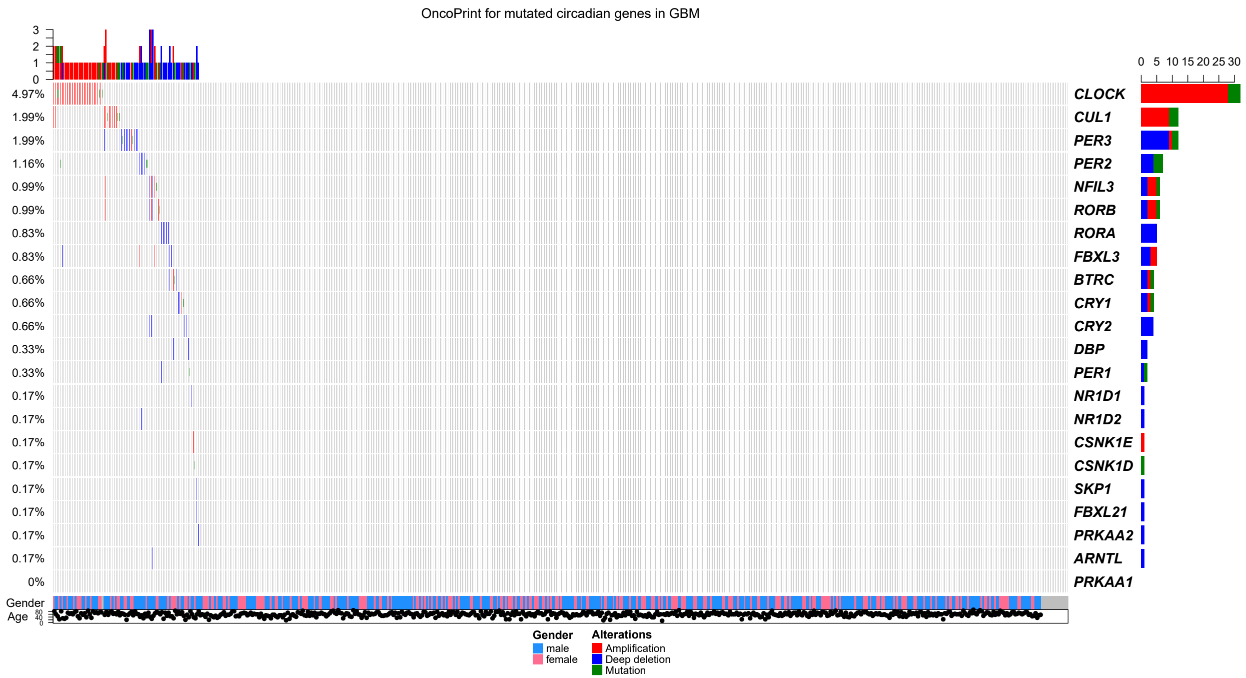

B

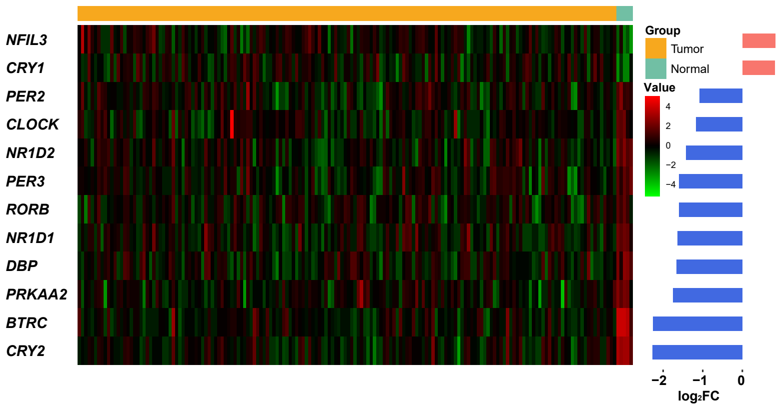

C

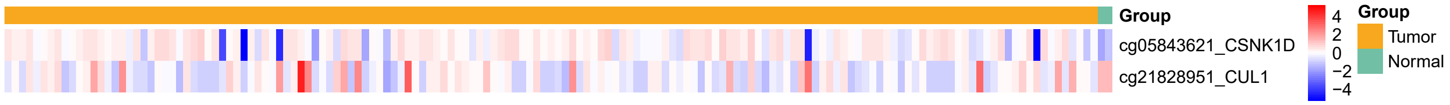

Figure S8H - Head and Neck squamous cell carcinoma (HNSC)

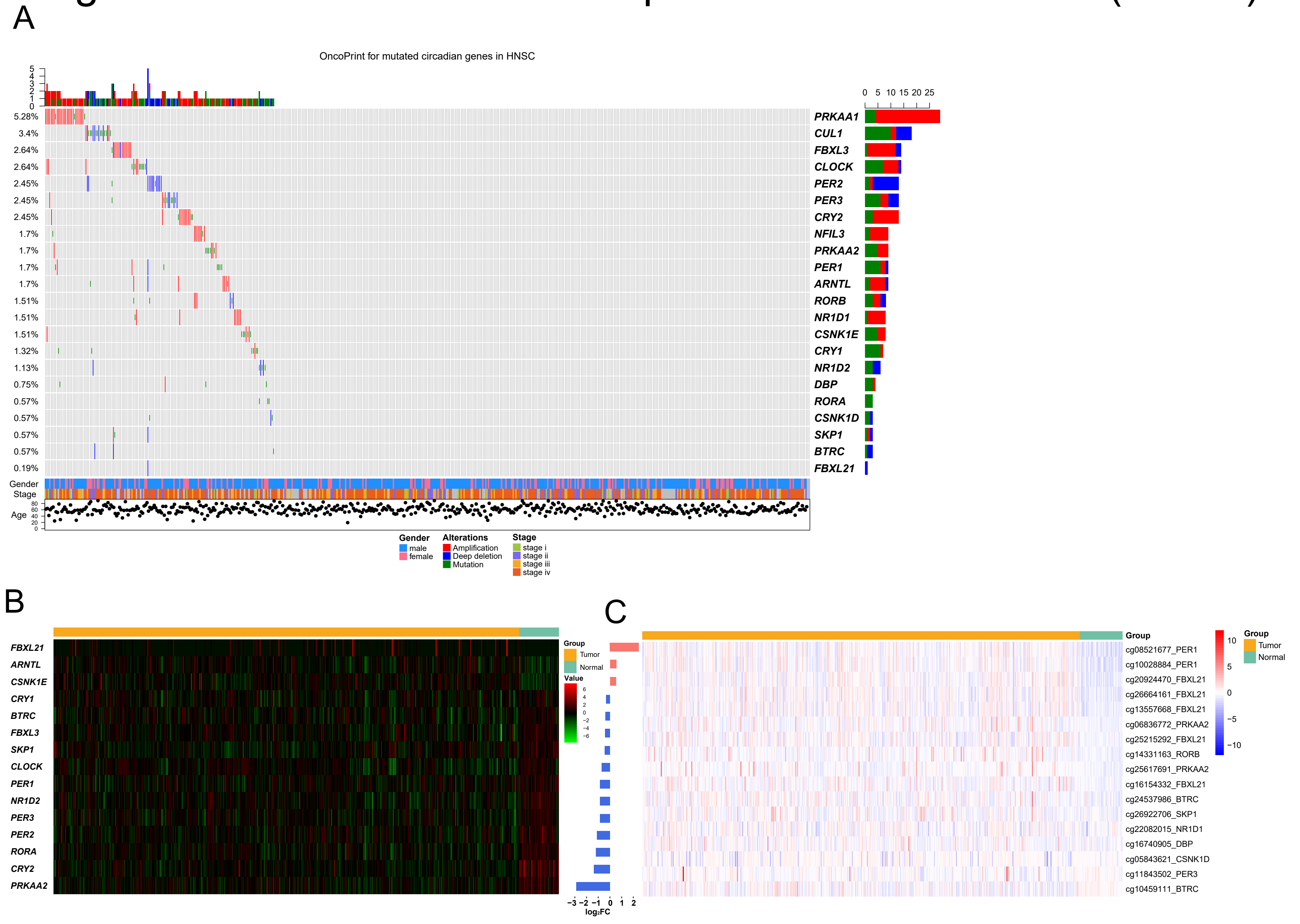

Figure S8I - Kidney Chromophobe (KICH)

A

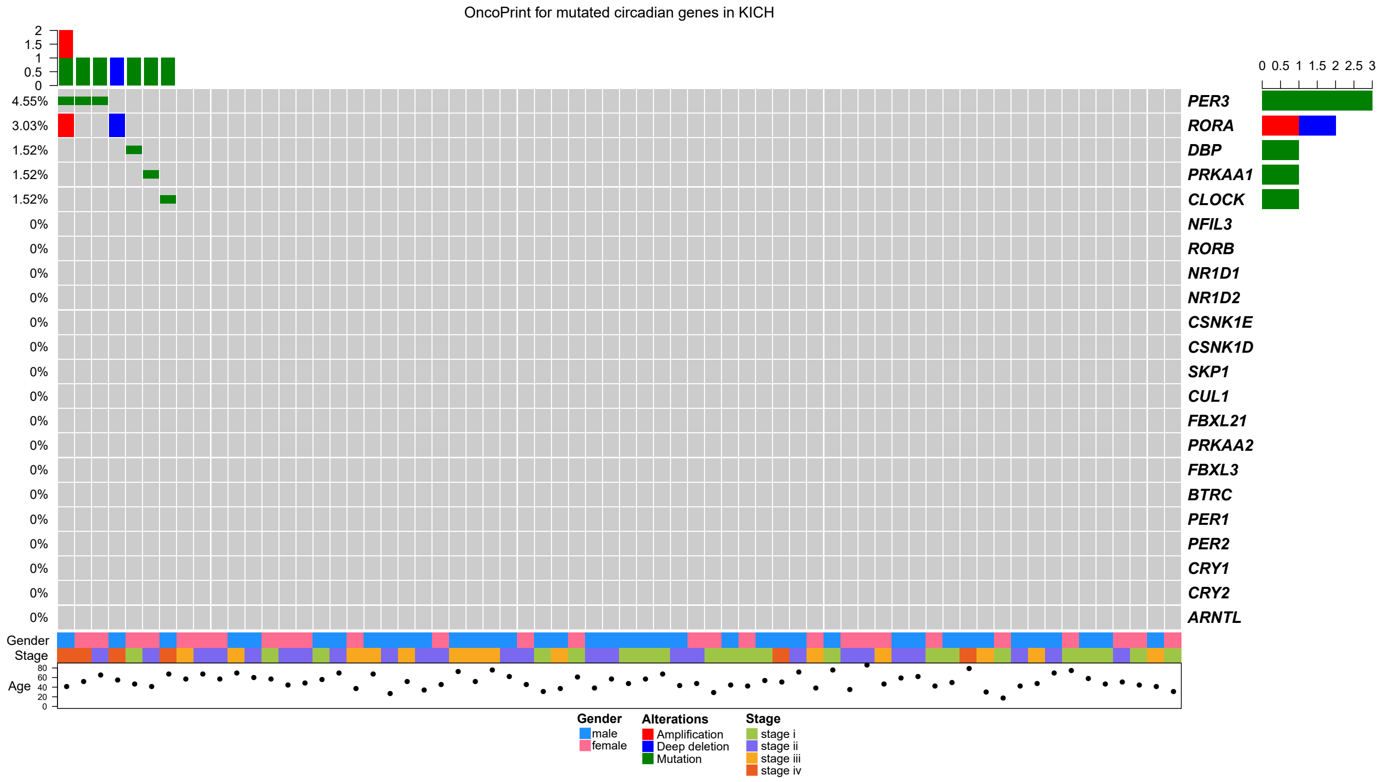

B

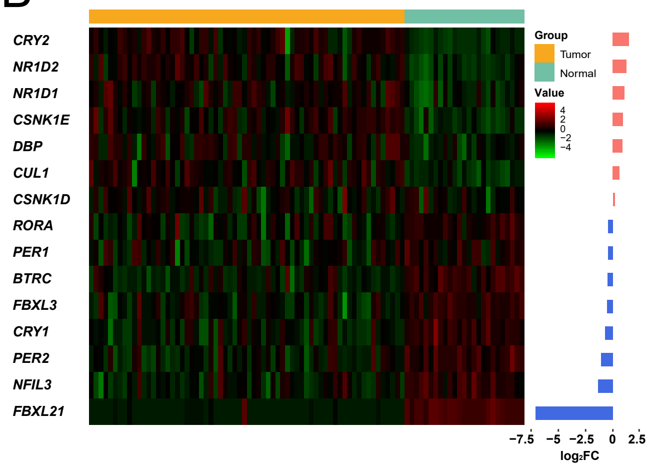

Figure S8J - Kidney renal clear cell carcinoma (KIRC)

A

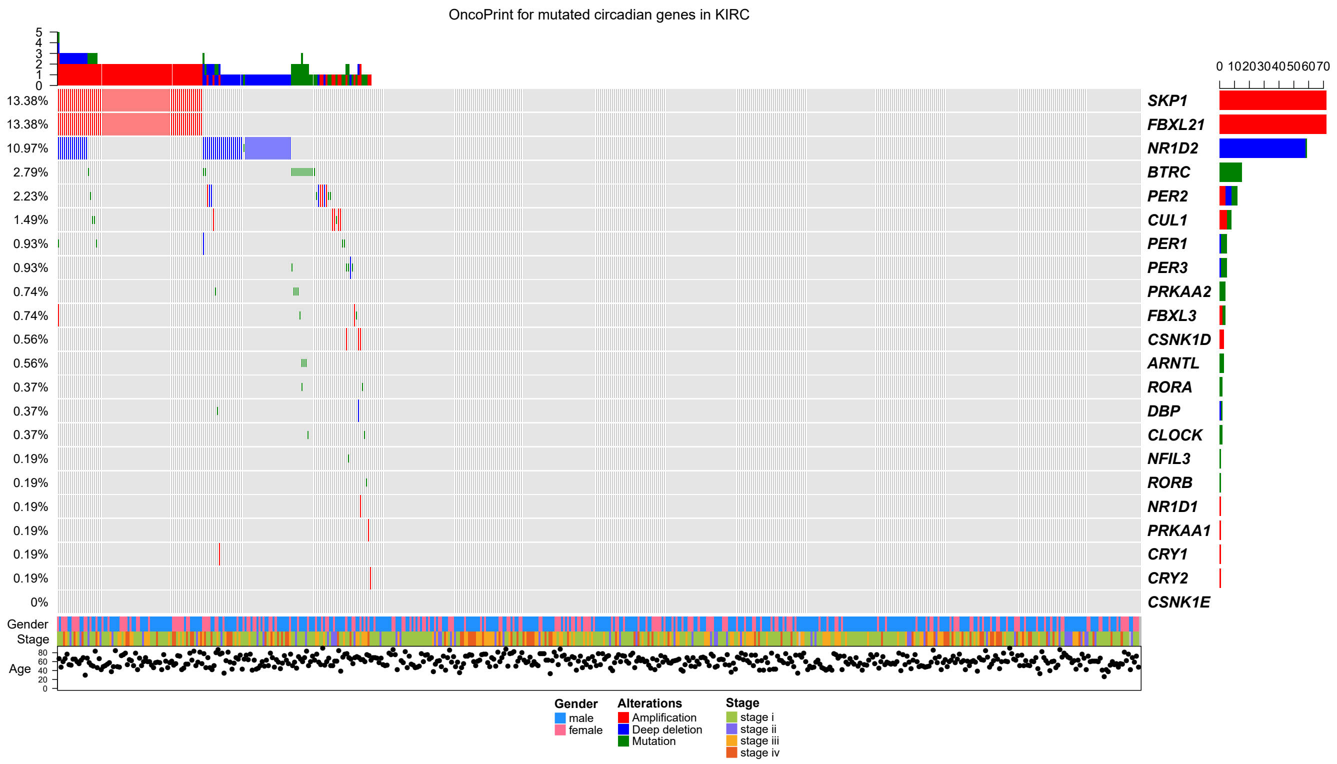

B

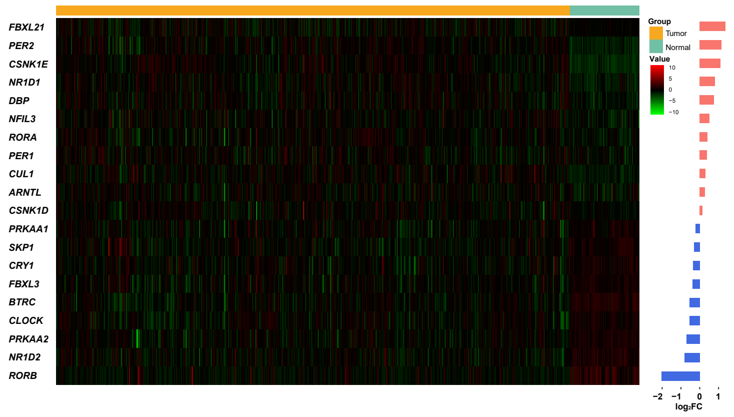

C

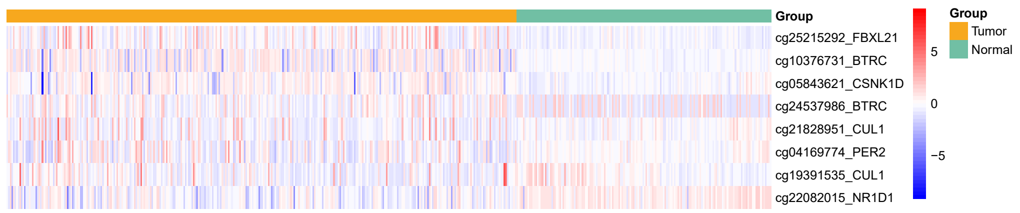

Figure S8K - Kidney renal papillary cell carcinoma (KIRP)

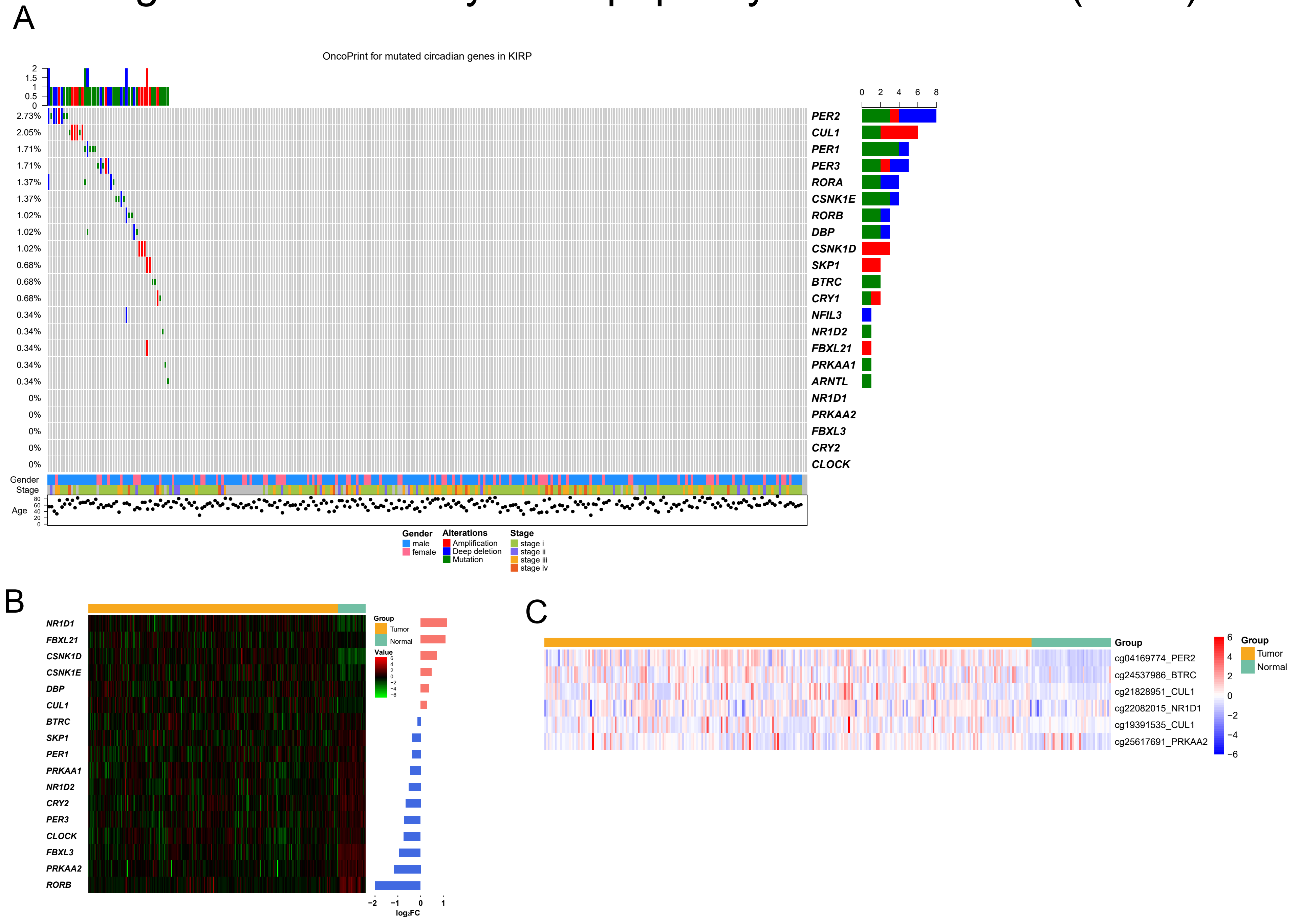

Figure S8L - Liver hepatocellular carcinoma (LIHC)

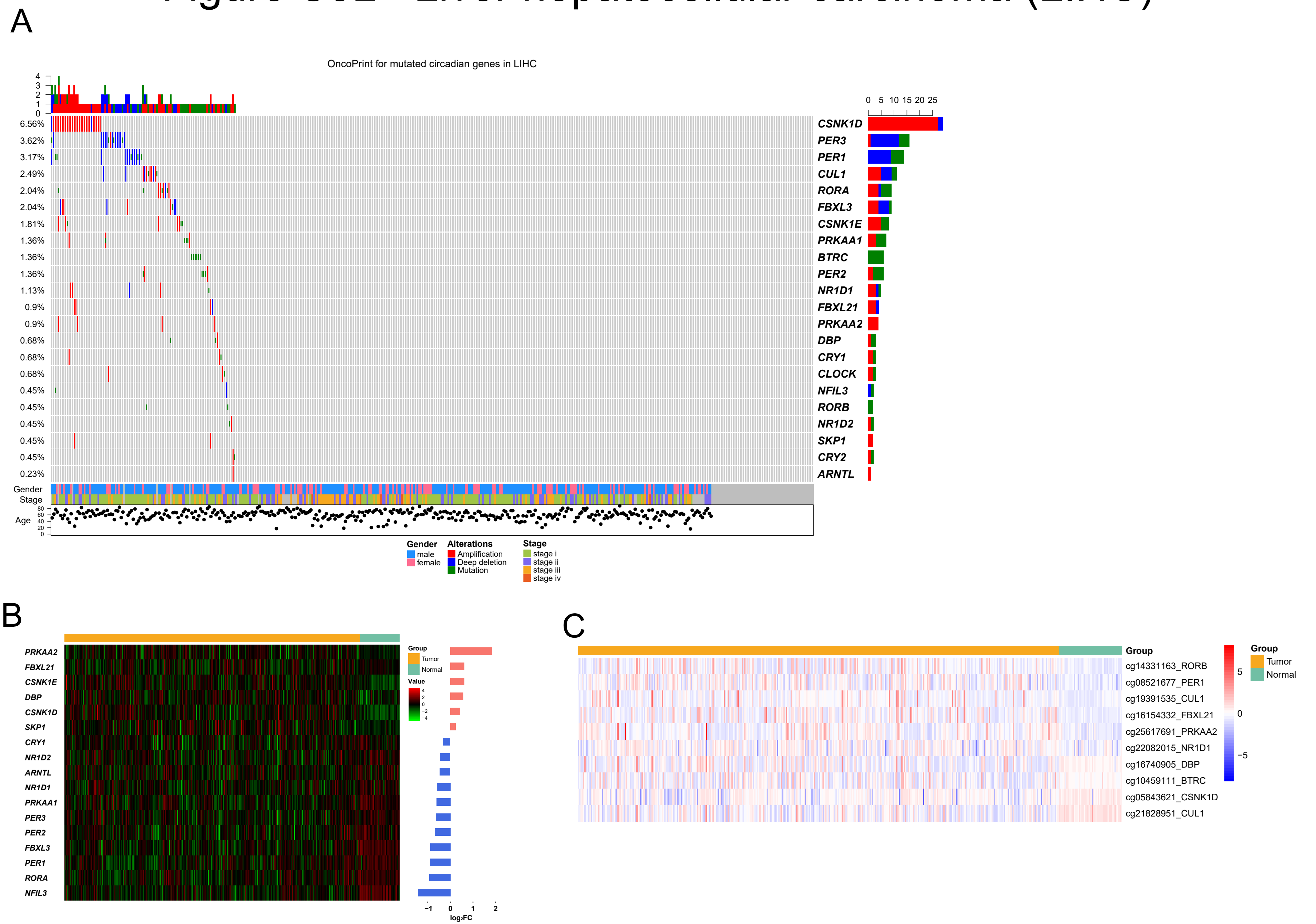

Figure S8M - Lung adenocarcinoma (LUAD)

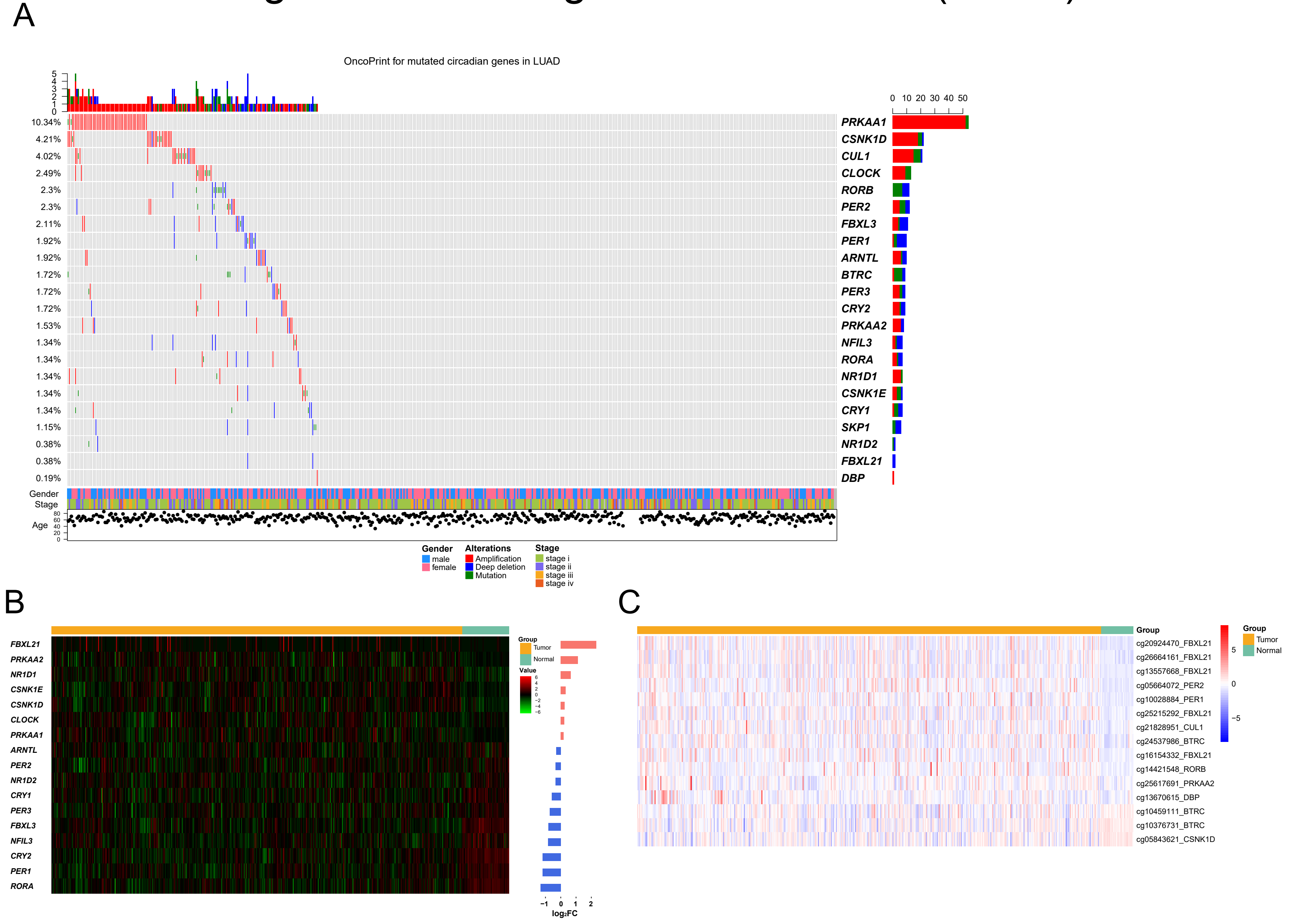

Figure S8N - Lung squamous cell carcinoma (LUSC)

A

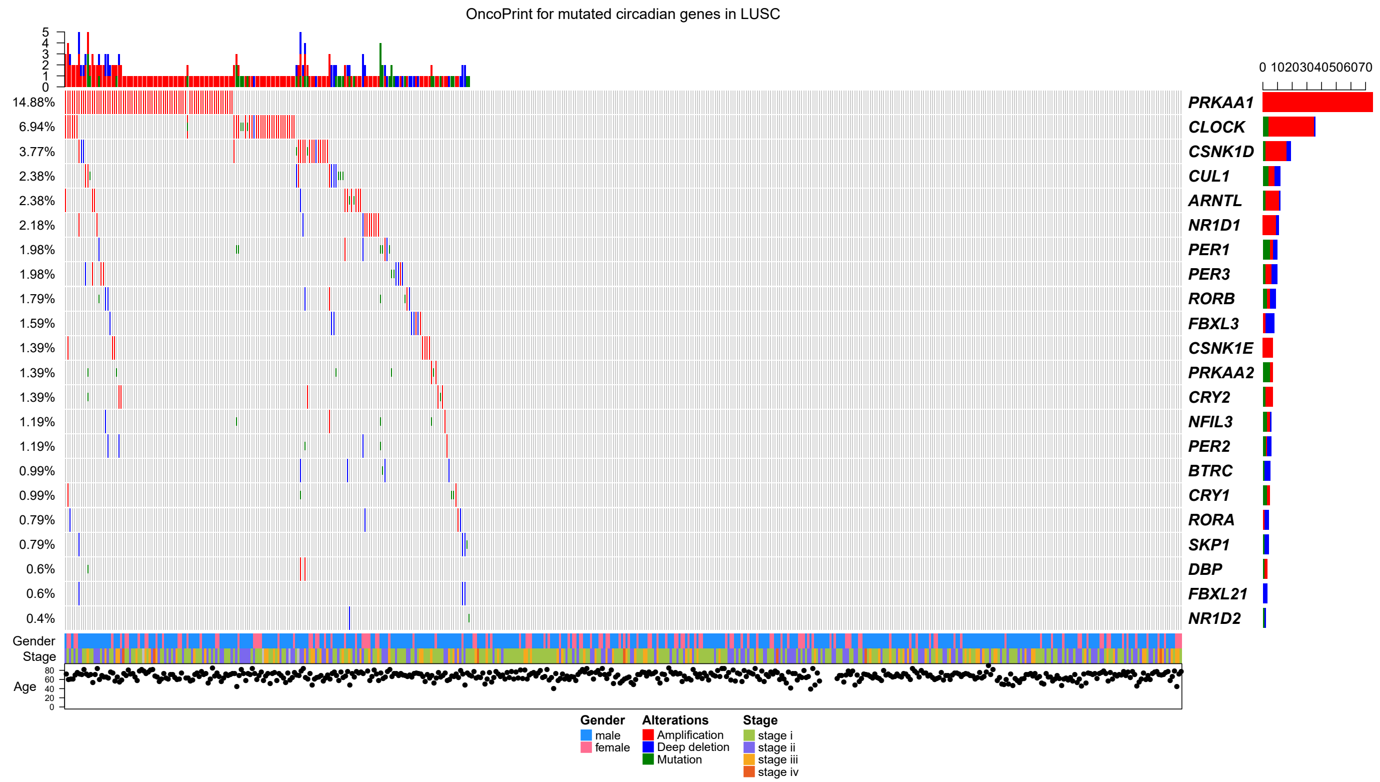

B

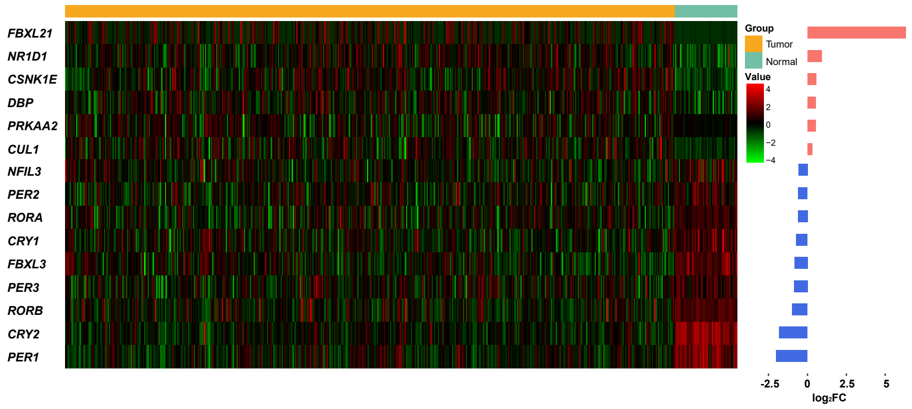

C

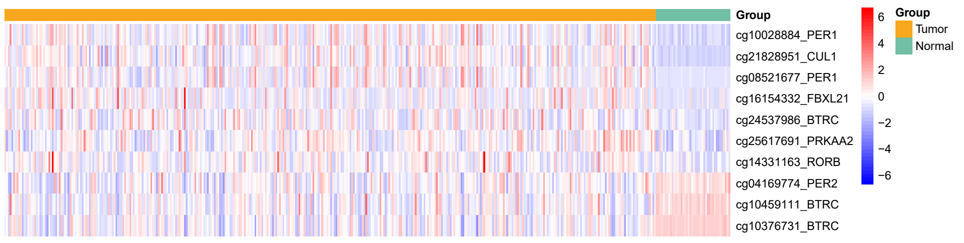

Figure S8O - Pancreatic adenocarcinoma (PAAD)

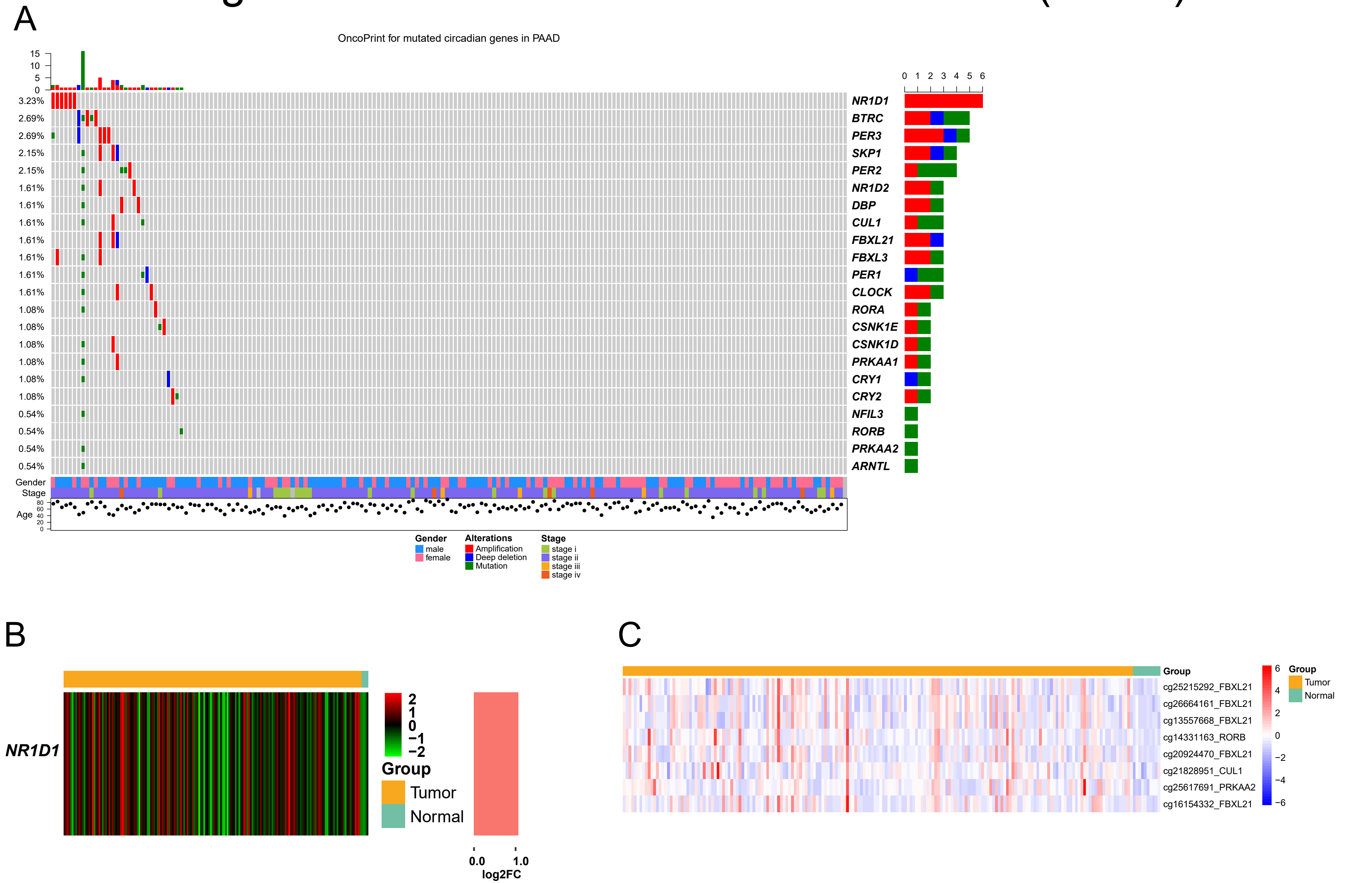

Figure S8P - Prostate adenocarcinoma (PRAD)

A

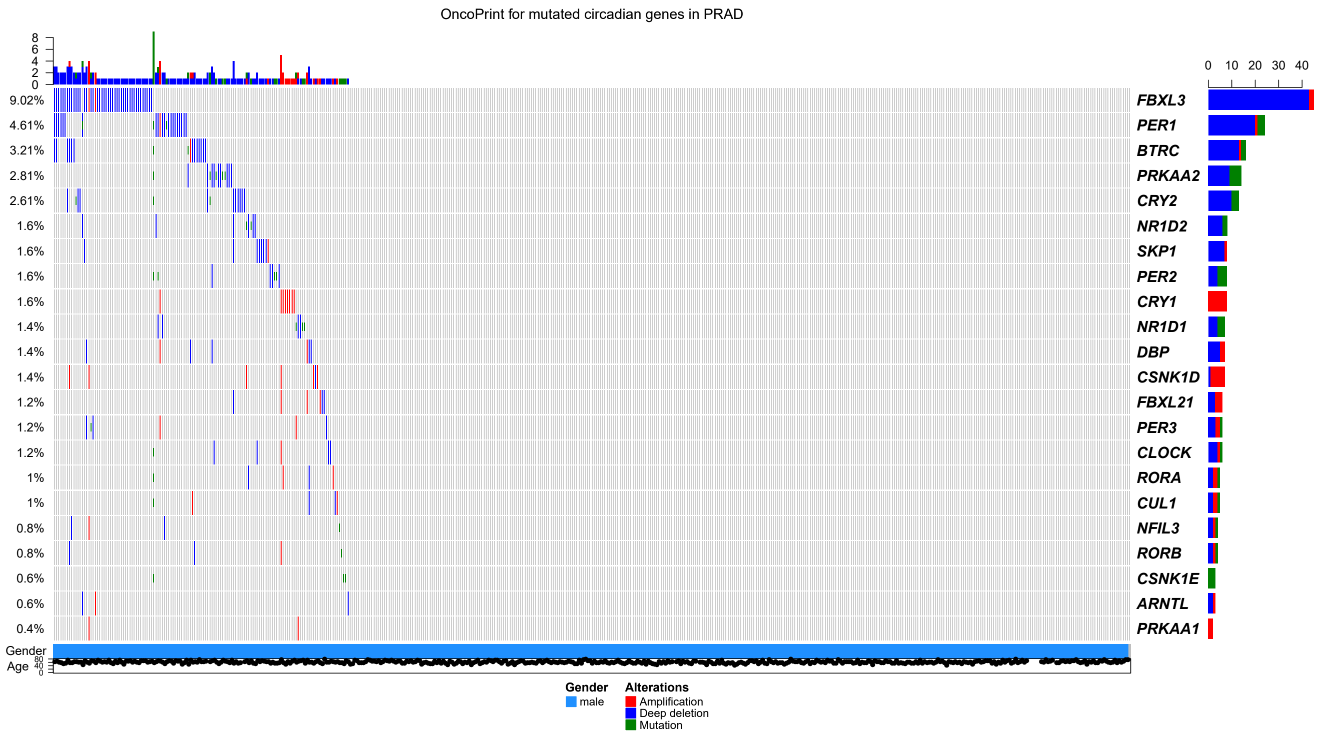

B

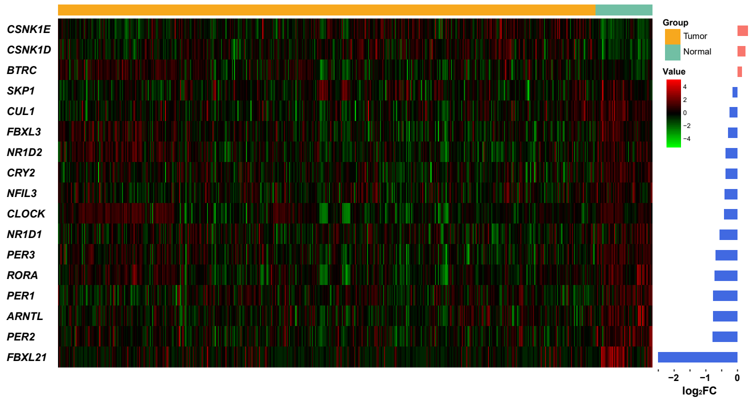

C

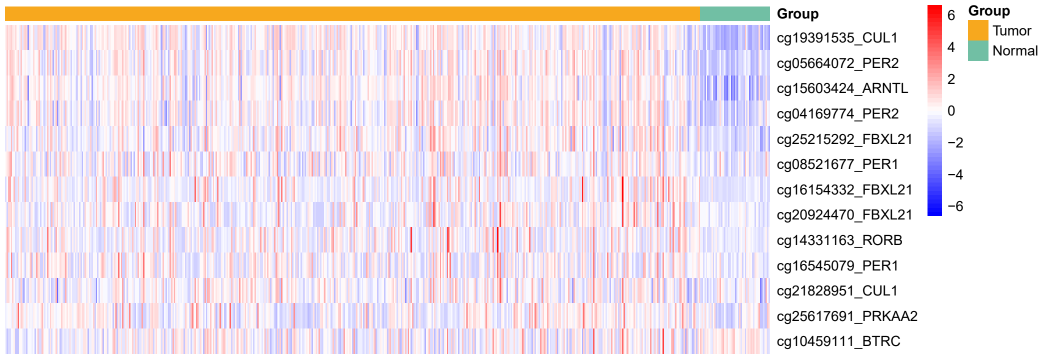

Figure S8Q - Rectum adenocarcinoma (READ)

A

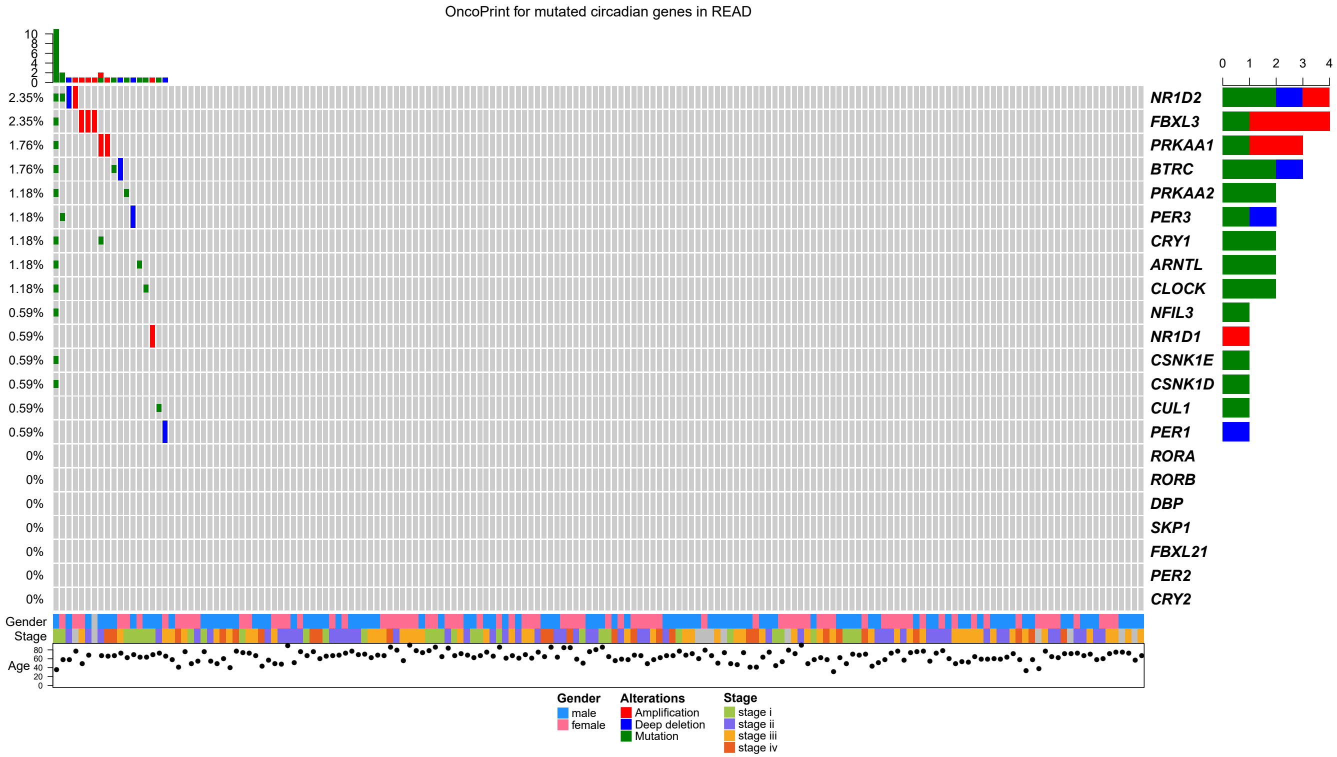

B

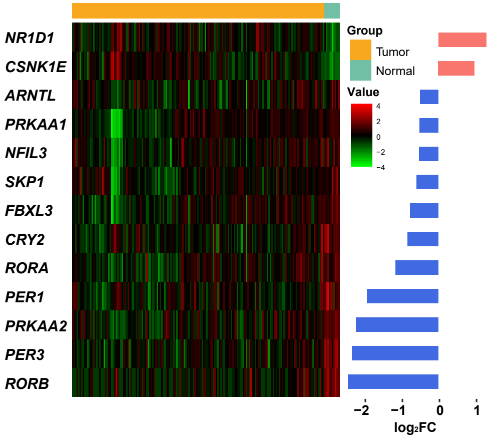

C

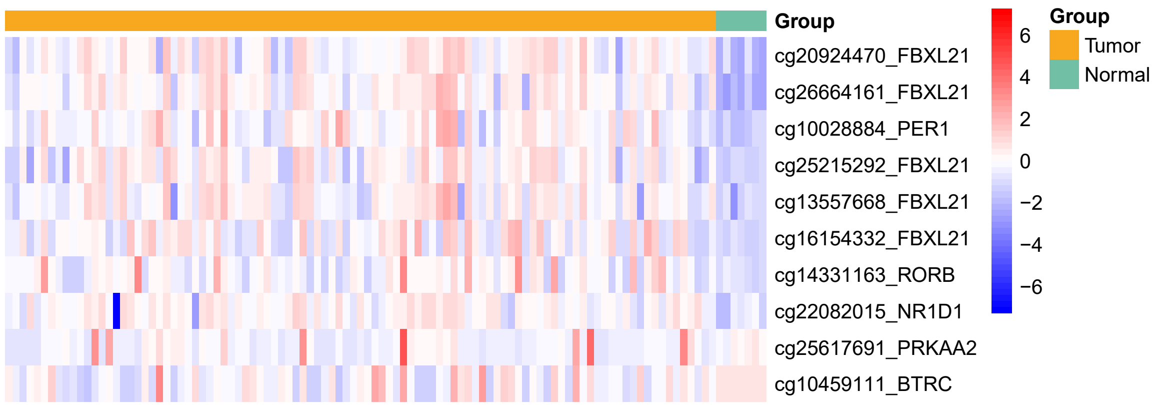

Figure S8R - Stomach adenocarcinoma (STAD)

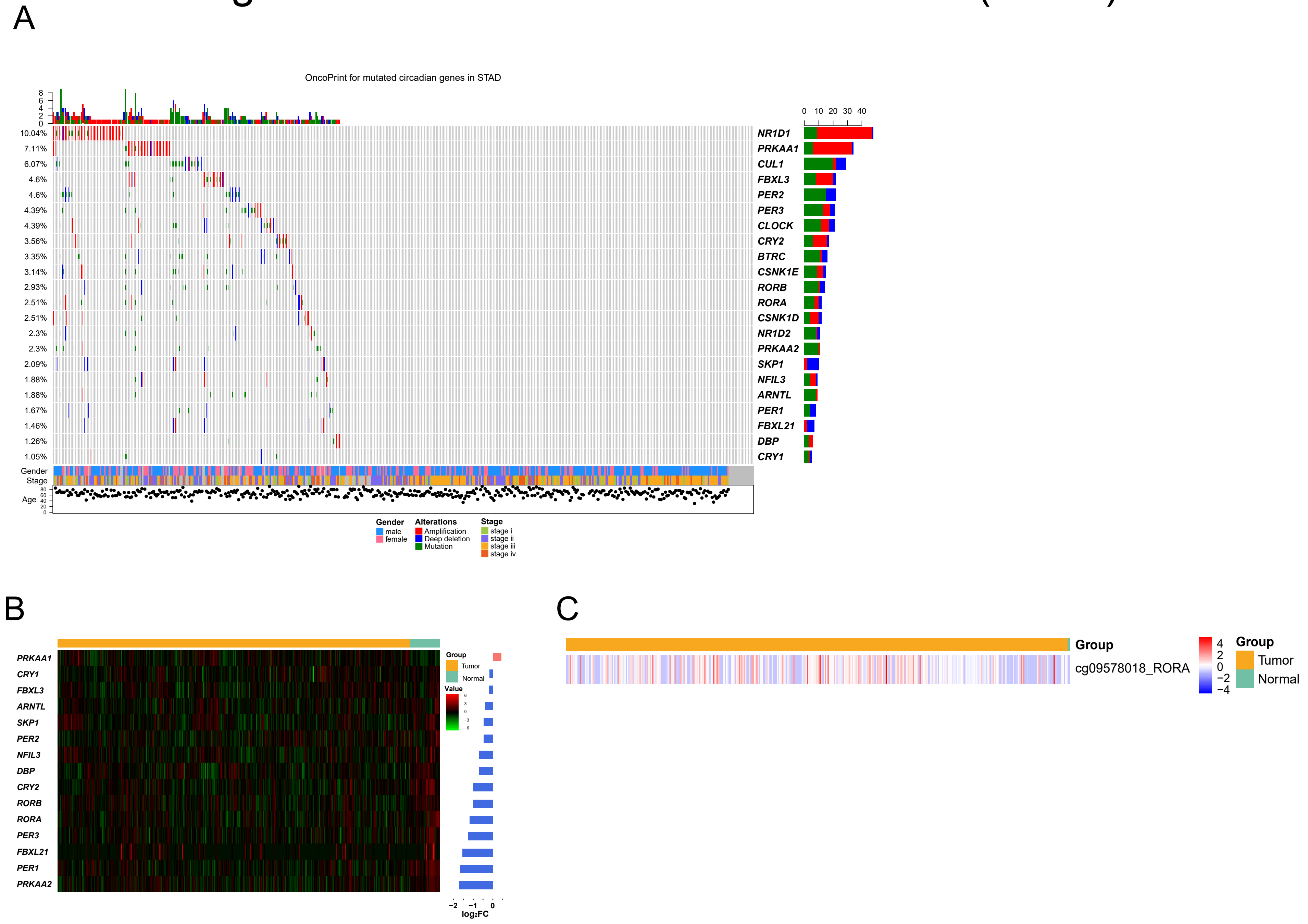

Figure S8S - Thyroid carcinoma (THCA)

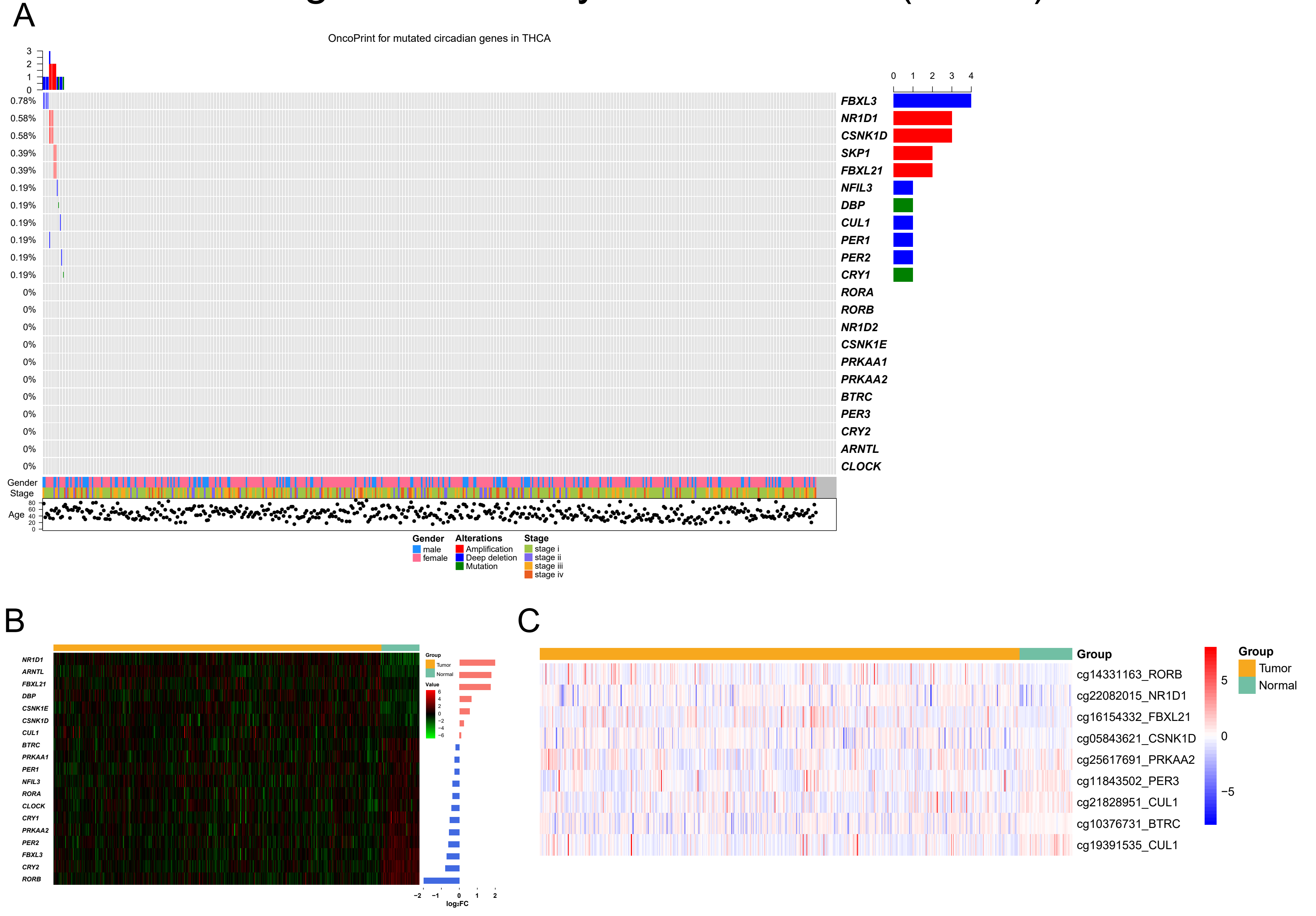

Figure S8T - Uterine Corpus Endometrial Carcinoma (UCEC)

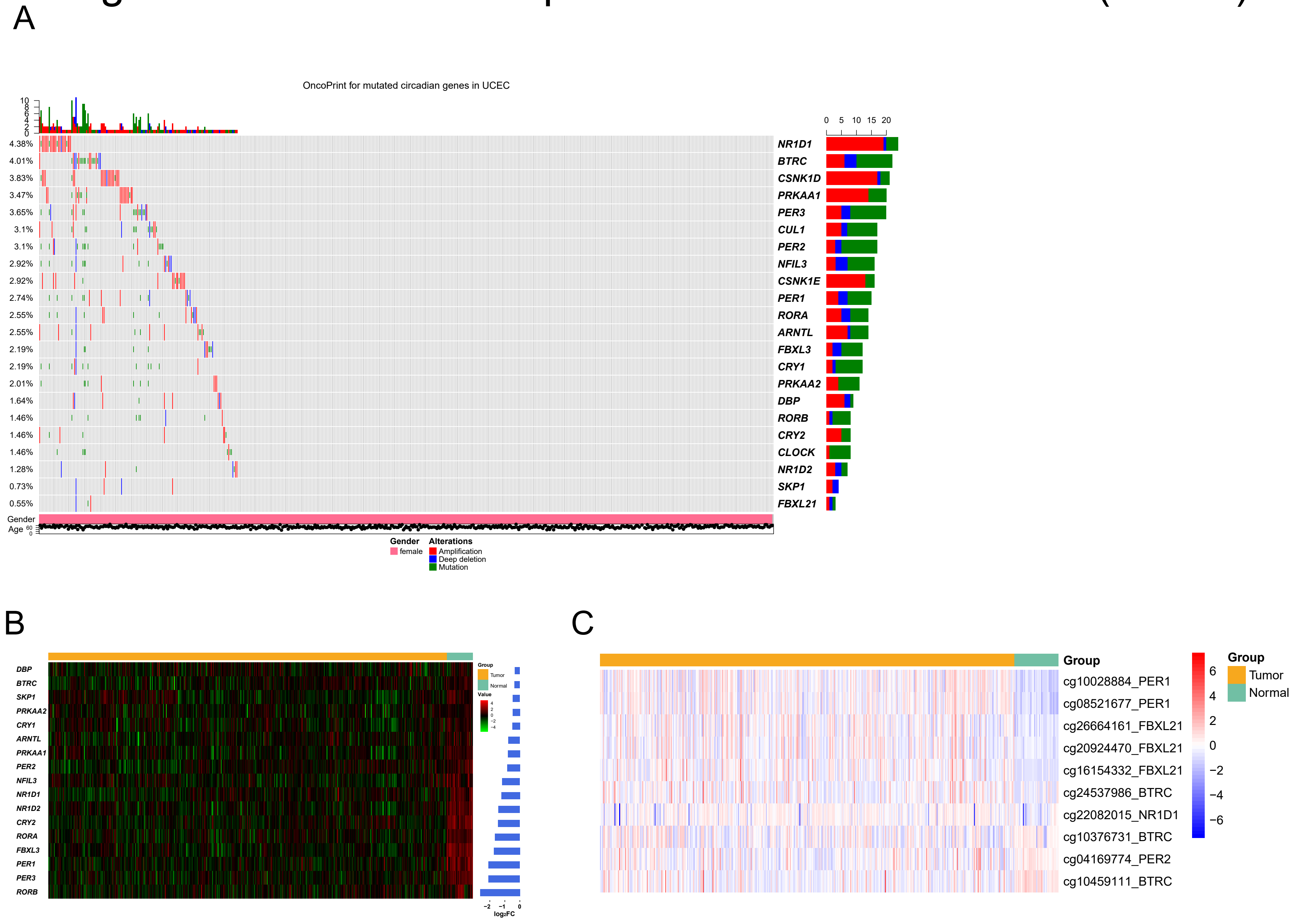

Supplement: Supplementary file 8 [file CAM4-8-1710-s008.pdf]
